# Development and validation of a novel combinatorial nomogram model to predict in-hospital deaths in heart failure patients

**DOI:** 10.1186/s12872-023-03683-0

**Published:** 2024-01-03

**Authors:** Dabei Cai, Qianwen Chen, Xiaobo Mu, Tingting Xiao, Qingqing Gu, Yu Wang, Yuan Ji, Ling Sun, Jun Wei, Qingjie Wang

**Affiliations:** 1 Department of Cardiology, the Affiliated Changzhou Second People’s Hospital of Nanjing Medical University, 29 Xinglong Alley, Changzhou, Jiangsu 213000 China; 2 Department of Anesthesiology, the Affiliated Wuxi People’s Hospital of Nanjing Medical University, Wuxi, Jiangsu 214023 China; 3 Department of Cardiovascular Surgery, the First Affiliated Hospital of Wannan Medical College, Wuhu, Anhui 241000 China; 4 Graduate School of Dalian Medical University, Dalian Medical University, Dalian, Liaoning 116000 China

**Keywords:** Heart failure, In-hospital death, Logistic regression, Nomogram, Medical information mart for intensive care

## Abstract

**Background:**

The purpose of this study was to develop a Nomogram model to identify the risk of all-cause mortality during hospitalization in patients with heart failure (HF).

**Methods:**

HF patients who had been registered in the Medical Information Mart for Intensive Care (MIMIC) III and IV databases were included. The primary outcome was the occurrence of all-cause mortality during hospitalization. Two Logistic Regression models (LR1 and LR2) were developed to predict in-hospital death for HF patients from the MIMIC-IV database. The MIMIC-III database were used for model validation. The area under the receiver operating characteristic curve (AUC) was used to compare the discrimination of each model. Calibration curve was used to assess the fit of each developed models. Decision curve analysis (DCA) was used to estimate the net benefit of the predictive model.

**Results:**

A total of 16,908 HF patients were finally enrolled through screening, of whom 2,283 (13.5%) presented with in-hospital death. Totally, 48 variables were included and analyzed in the univariate and multifactorial regression analysis. The AUCs for the LR1 and LR2 models in the test cohort were 0.751 (95% CI: 0.735∼0.767) and 0.766 (95% CI: 0.751–0.781), respectively. Both LR models performed well in the calibration curve and DCA process. Nomogram and online risk assessment system were used as visualization of predictive models.

**Conclusion:**

A new risk prediction tool and an online risk assessment system were developed to predict mortality in HF patients, which performed well and might be used to guide clinical practice.

**Supplementary Information:**

The online version contains supplementary material available at 10.1186/s12872-023-03683-0.

## Introduction

Heart failure (HF), a disorder in which systolic or diastolic dysfunction of the heart is attributed to structural or functional abnormalities of the heart [1]. HF will be the final stage of development of various heart diseases [2]. Approximately 40 million people worldwide suffer from HF [3]. Currently, in Europe, the prevalence of HF is approximately 3/1000 person-years (all ages) or approximately 5/1000 person-years in adults [4, 5]. In the United States, more than 5 million people are living with HF, and the number continues to increase at a rate of 550,000 cases diagnosed each year [6, 7]. At the same time, there are about 8.9 million HF patients in China, and the prevalence rate of those over 35 years old is 1.3% [8]. The prevalence of HF increases with age: from about 1% in those < 55 years old to > 10% in those 70 years or older [9–11]. HF, as part of cardiovascular disease and a major public health problem worldwide, is an important cause of rising global mortality [12]. The annual direct and indirect costs of HF are estimated at $29 billion due to its high prevalence, poor prognosis, and high readmission rates [13]. In clinical practice, simple yet effective tools play a key role in predicting future events, especially in making decisions about primary prevention and treatment of HF patients. Therefore, effective mortality prediction can help doctors formulate more scientific treatment plans to prevent its deterioration, thereby improving the quality of life and reducing medical expenses.

The Nomogram is used as a graphical device that integrates predictors to determine the probability of a clinical event occurring in a given patient [14]. The Nomogram is based on a logistic regression (LR) model that integrates multiple clinical predictors and displays these individual predictor contribution scores to accurately predict an individual patient’s risk of a clinical event, helping clinicians to optimize individualized treatment choices and assess treatment outcomes [15–21].

The aim of this study was to develop and validate robust risk assessment models to predict all-cause mortality during hospitalization in HF patients. And to develop Nomogram and develop an online risk assessment system.

## Methods

### Data source

HF patient data was obtained from the Medical Information Marketplace in Intensive Care (MIMIC) III and MIMIC-IV databases. MIMIC-III contains data associated with 53,423 distinct hospital admissions for adult patients (aged 16 years or above) admitted to critical care units between 2001 and 2012. In addition, it contains data for 7870 neonates admitted between 2001 and 2008. The data covers 38,597 distinct adult patients and 49,785 hospital admissions [22]. The MIMIC-IV database covers information on all patients at Beth Israel Deaconess Medical Center who recorded 523,740 admissions between 2008 and 2019, of which 76,540 were admitted to the ICU for admission [23]. Clinical records including demographic data, vital signs, laboratory test results, microbiological culture results, imaging data, treatment regimens, medication records, and survival information are recorded in the MIMIC database. Use of the MIMIC database has been approved by the Beth Israel Deaconess Medical Center and the MIT Review Board. We received permission after applying for and completing the course and testing (Record Nos. 44703031 and 44703032). Informed consent was not required as all patient information in the database is anonymized [24, 25].

### Patients enrollment and data collection

Data were extracted using SQL (Structured Query Language) programming in Navicat Premium (version 15.0.12). Ninth revision of the International Classification of Diseases (ICD-9/10) codes were used to identify all patients hospitalized for congestive HF. Exclusion criteria: 1) patients younger than 18 years or older than 90 years; 2) patients with more than 20% missing data were excluded from the analysis. When patients are older than 90 years, these patients will be assigned an age of 300 years in MIMIC III and 91 years in MIMIC IV. Their actual age is unknown. We assigned the MIMIC-IV data to the training cohort for model building in the training cohort. The MIMIC-III patient data were used to perform the validation function of the model.

After identifying eligible subjects, we collected clinical data including demographics, comorbidities, vital signs, and laboratory parameters. Comorbidities included atrial fibrillation (AF), previous myocardial infarction (p-MI), type 2 diabetes mellitus (T2DM), hypertension, ventricular arrhythmias (VA), and acute kidney injury (AKI). Vital signs were collected from the first recorded results at the time of hospitalization and included heart rate (HR), respiratory rate (RR), temperature (T), Systolic blood pressure (SBP), Diastolic blood pressure (DBP), and mean artery pressure (MAP). Post-admission laboratory parameters were also obtained for the first time. The indicators studied were red blood cells (RBC), white blood cells (WBC), platelets, hemoglobin, hematocrit, mean red blood cell volume (MCV), mean red blood cell hemoglobin volume (MCH), mean red blood cell hemoglobin concentration (MCHC), albumin, alanine aminotransferase (ALT), aspartate Transaminase (AST), total bilirubin (TB), alkaline phosphatase (AP), and blood urea nitrogen (BUN), creatinine, glucose, lactate, total carbon dioxide (T-CO2), arterial partial pressure of oxygen (PaO_2_), arterial carbon dioxide partial pressure, (PaCO_2_) arterial oxygen saturation (SaO_2_), potential of hydrogen (pH), anion gap (AG), base excess (BE), bicarbonate, potassium, sodium, chloride, total calcium (T-calcium), phosphorus, magnesium, activated partial thromboplastin time (APTT), prothrombin time (PT), international normalized ratio (INR).

The diagnosis of AKI is based on the latest International Clinical Practice Guidelines for AKI [26]. Any of the following three criteria meet the diagnostic criteria. (a) increase in creatinine by ≥ 0.3 mg/dl (≥ 26.5 μmol/L) within 48 h; (b) increase in creatinine to ≥ 1.5 times baseline, which is known or presumed to have occurred within the prior 7 days; (c) urine volume < 0.5 ml/kg/h for 6 h. Patients with CKD stage 5 will be excluded from AKI even if they meet the above criteria. In-hospital AKI diagnoses can also be accessed directly through the officially provided view codes. Hospitalization numbers for ICDs documenting paroxysmal ventricular tachycardia, ventricular flutter, and ventricular fibrillation will be flagged as VA.

### Model construction and evaluation

LR models were used for model construction. Nomogram was used to visualize the regression model [27]. Calibration curves can be used as one of the evaluation indicators of the model to assess the goodness of fit of the model [28]. Decision curve analysis (DCA) can demonstrate the net benefit of an intervention by estimating the clinical utility of a predictive model based on a threshold probability (the probability of triggering a medical intervention by a physician or patient, corresponding to the probability that the harm of a false-positive intervention exceeds the harm of a false-negative no intervention) [29, 30]. Once the model was established, data from the test cohort and validation cohort were used to further evaluate the performance of the model. Area and precision-recall curves under the receiver operating characteristic curve (AUC) were used to compare the performance of each model. We also calculated the net reclassification improvement (NRI) and integrated discrimination improvement (IDI) to evaluate the improvement of the new models [31, 32].

### Study endpoint

The endpoint event is in-hospital all-cause mortality; patients whose date of death coincides with the date of discharge or is less than 12 h from the date of discharge will be defined as having experienced in-hospital death.

### Statistical analysis

During the data collection phase, every laboratory test result during the patient’s hospitalization will be collected and composed in a huge raw table. At this point, all variables were collated and, to avoid excessive bias. Variables with less than 20% missing values are randomly filled in using multiple interpolation, which is based on the R package “mice”. The missing proportions of all continuous variables before filling are displayed in the (Supplementary Table 1, Supplementary Figs. 1 and 2). Finally, in chronological order, only the results of the patient’s first laboratory examination were retained for the subsequent study.

Categorical variables were described by frequencies and percentages, and differences between groups were determined by chi-square test or Fisher’s exact test. Continuous variables were expressed as mean ± standard deviation or median and interquartile range (IQR), and groups were compared using Student’s t-test or Mann–Whitney u-test.

Univariate LR analyses were first performed, and variables with a probability of inclusion < 0.05 were selected for multivariate LR analysis. Those variables that still had an independent effect on outcome after multivariate correction would be retained. When their *P*-value is less than 0.001 will be used as predictor variables to develop the model.

The first LR (LR1) model was developed, which was incorporated with all continuous variables whose *P*-values remained less than 0.001 after multifactorial adjustment. predictors of the LR1 model included: age, RR, PaO_2_, platelet count, albumin, TB, AP, lactate, pH, BE, and phosphorus.

A second LR model (LR2) was developed by adding the variables AKI and VA to the LR1 model. R software (version 4.2.1) was used for statistical analysis; GraphPad Prism (version 8.3.0) was used to draw graphs; and *P* < 0.05 was considered statistically significant.

## Results

### Baseline characteristics

**Fig. 1:**
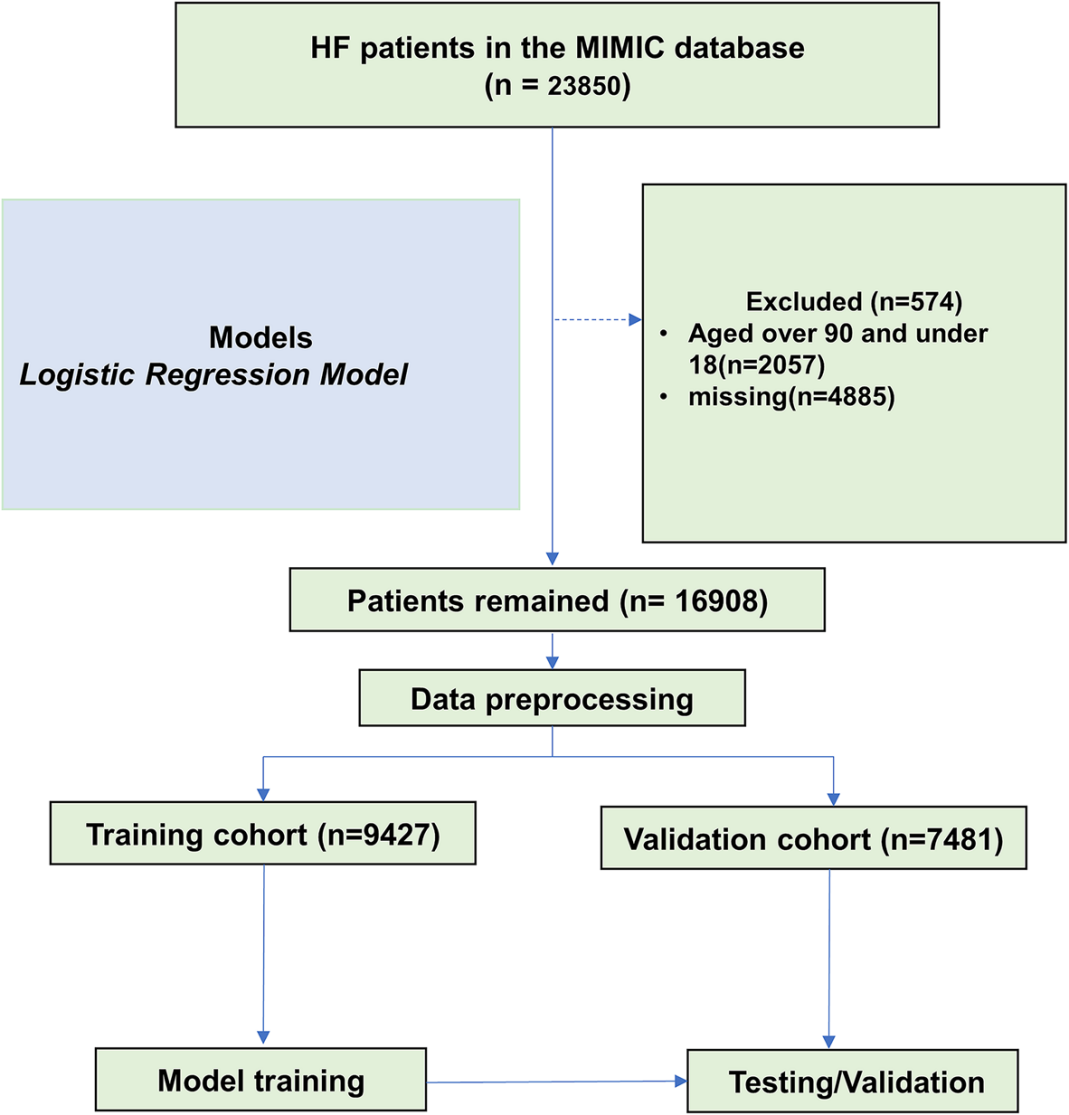
Flow diagram of the selection process of patients

A total of 16,908 patients were included in this study, including 7,481 in the MIMIC III cohort and 9,427 in the MIMIC IV cohort (Fig. 1). Cumulative in-hospital deaths were 2283 (13.5%, Supplemental Table 2). In-hospital deaths occurred in a total of 1075 (14.4%) patients in the MIMIC III cohort; in-hospital deaths occurred in a total of 1208 (12.8%) patients in the MIMIC IV cohort, a significant difference (*P* = 0.004, Supplemental Table 3). Compared with the surviving cohort, those who died had a higher mean age, higher mean heart rate, and faster RR; lower arterial systolic, diastolic, and mean arterial pressures, and lower median body temperature levels (Supplementary Table 2). In the MIMIC III cohort, a total of 1075 (14.4%) patients experienced in-hospital death, with a higher mean age in the death group (Table 1). The death group had higher HR, RR and temperature, lower blood pressure, lower rates of combined hypertension and T2DM, and higher rates of combined AKI and VA (Table 1). There were no significant differences in other comorbidities. In the MIMIC IV cohort, a total of 1208 (12.8%) patients died in-hospital, and the mean age of the death group was higher than that of the survivor group (Supplementary Table 4). Compared with the survivor group, patients in the death group had faster HR, RR, higher temperature, lower blood pressure, lower rates of comorbid hypertension, and higher rates of AKI and VA. There were also no significant differences in other co-morbidities (Supplementary Table 4).

**Table 1:** Baseline characteristics

	**MIMIC III (*N* = 7481)**		
	**Survival (*n* = 6406)**	**Dead (*n* = 1075)**	***P-value***
**Demographic**			
Age (Year)	69.91 (13.05)	73.12 (12.57)	< 0.001
Male (n%)	3445 (53.8)	558 (51.9)	0.269
**Vital signs**			
HR (minˉ^1^)	88.81 (19.31)	93.22 (22.05)	< 0.001
RR (minˉ^1^)	18.92 (6.17)	21.26 (6.76)	< 0.001
SBP (mmHg)	122.47 (23.63)	118.15 (27.74)	< 0.001
DBP (mmHg)	63.73 (14.97)	62.09 (15.62)	0.001
MAP (mmHg)	83.31 (15.53)	80.78 (17.05)	< 0.001
T (°C)	36.60 (0.86)	36.61 (1.02)	0.642
**Laboratory results**			
RBC (m/uL)	3.90 (0.75)	3.80 (0.75)	< 0.001
WBC (k/uL)	10.20 [7.50,13.80]	11.30 [7.75,15.65]	< 0.001
Platelet (k/uL)	219.00 [170.00,273.00]	209.00 [140.00,265.50]	< 0.001
Hemoglobin (g/dL)	11.64 (2.23)	11.33 (2.13)	< 0.001
Hematocrit (%)	34.81 (6.41)	34.23 (6.34)	0.006
MCV (fL)	89.56 (6.61)	90.72 (7.46)	< 0.001
MCH (pg)	30.00 (2.61)	30.05 (2.92)	0.51
MCHC (%)	33.50 (1.55)	33.12 (1.64)	< 0.001
Albumin (mg/dL)	3.16 (0.42)	2.93 (0.55)	< 0.001
ALT (IU/L)	38.00 [19.00, 88.00]	37.00 [19.00, 88.00]	0.308
AST (IU/L)	49.00 [25.00, 117.00]	58.00 [27.00, 117.00]	< 0.001
TB (mg/dL)	0.90 [0.50,1.90]	0.90 [0.50,1.90]	0.024
AP (IU/L)	107.00 [71.00,130.00]	112.00 [74.00,134.00]	< 0.001
BUN (mg/dL)	23.00 [16.00,33.00]	31.00 [20.00,48.00]	< 0.001
Creatinine (mg/dL)	1.30 [1.10,1.50]	1.40 [1.20,1.85]	< 0.001
Glucose (mg/dL)	133.00 [108.00,173.00]	135.00 [105.00,183.00]	0.746
Lactate (mg/dL)	2.40 [1.40,2.50]	2.30 [1.50,3.55]	< 0.001
T-CO_2_ (mEq/L)	25.00 [25.00,29.00]	25.00 [20.00,28.00]	< 0.001
pH (units)	7.38 (0.08)	7.34 (0.12)	< 0.001
PaO_2_ (mmHg)	105.56 (25.12)	96.02 (25.41)	< 0.001
SaO_2_ (%)	95.47 (6.52)	94.40 (7.52)	< 0.001
PaCO_2_ (mmHg)	43.15 (10.05)	42.83 (13.73)	0.362
AG (mEq/L)	15.01 (3.64)	16.42 (4.21)	< 0.001
BE (mEq/L)	0.00 [0.00,2.00]	0.00 [-5.00,2.00]	< 0.001
Bicarbonate (mg/dL)	24.86 (5.08)	23.61 (5.73)	< 0.001
Potassium (mEq/L)	4.33 (0.78)	4.47 (0.87)	< 0.001
Sodium (mEq/L)	138.02 (4.40)	137.48 (5.63)	< 0.001
Chloride (mEq/L)	102.31 (5.88)	101.57 (6.56)	< 0.001
T-Calcium (mEq/L)	8.60 (0.77)	8.46 (0.90)	< 0.001
Magnesium (mg/dL)	1.97 (0.38)	2.00 (0.42)	0.05
Phosphate (mg/dL)	3.60 [3.00,4.20]	3.90 [3.10,4.70]	< 0.001
INR	1.30 [1.10,1.60]	1.40 [1.20,1.90]	< 0.001
APTT (s)	30.20 [26.20,36.50]	31.50 [26.90,38.85]	< 0.001
PT (s)	14.10 [13.00,16.30]	14.80 [13.25,18.20]	< 0.001
**Comorbidities (n%)**			
AF	2670 (41.7)	478 (44.5)	0.093
T_2_DM	1636 (25.5)	220 (20.5)	< 0.001
Hypertension	3201 (50.0)	427 (39.7)	< 0.001
p-MI	602 (9.4)	74 (6.9)	0.009
VA	563 (8.8)	130 (12.1)	0.001
AKI	2131 (33.3)	597 (55.5)	< 0.001
CLD	2277 (35.5)	393 (36.6)	0.544
MT	330 (5.2)	68 (6.3)	0.13
CKD	4988 (77.9)	818 (76.1)	0.211
Anemia	2438 (38.1)	414 (38.5)	0.803

Values are mean + SD, n (%), or median (IOR)

*HR* heart rata, *RR* Respiratory rate, *SBP* systolic blood pressure, *DBP* diastolic blood pressure, *MAP* mean arterial pressure, *T* Temperature; arterial oxygen saturation, *RBC* red blood cell, *WBC* white blood cell, *MCV* mean corpuscular volume, *MCH* mean corpuscular hemoglobin, *MCHC* mean corpuscular hemoglobin concentration, *ALT* aspartate aminotransferase, *AST* aspartate aminotransferase, *TB* Total Bilirubin, *AP* Alkaline phosphatase, *BUN* blood urea nitrogen, *T-CO*_2_ Total carbon dioxide, *pH* potential of hydrogen, *PaO*_2_ arterial partial pressure of oxygen, *PaCO2* arterial partial pressure of carbon-dioxide, SaO_2_, *AG* anion gap, *BE* base excess, *INR* International Normalized Ratio, *PT* prothrombin time, *APTT* activated partial prothrombin time, *AF* atrial fibrillation, *T2DM* type 2 Diabetes Mellitus, *p-MI* previous myocardial infarction, *VA* ventricular arrhythmias, *AKI* acute kidney injury, *CLD* chronic lung disease, *MT* malignant tumor, *CKD* chronic kidney diseases

### Feature selection and regression analysis

Data from the MIMIC III and MIMIC IV cohorts will be combined for univariate and multivariate LR analyses on in-hospital all-cause mortality. After all variables were analyzed by univariate binary LR, those variables with *P*-value still less than 0.05 will be included in the multifactorial binary LR analysis for adjustment. Ultimately, we found that age, albumin, Sodium, bicarbonate, lactate, magnesium, phosphate, platelets, AG, T-CO_2,_ MCV, HR, PaO_2_, AP, BE, RBC, RR, TB, WBC, pH, the occurrence of AKI, and the occurrence of VA were independently associated with the occurrence of in-hospital mortality in HF patients. The dominance ratio odds ratio (OR) and 95% confidence interval (95% CI) were calculated for the predictors of in-hospital all-cause mortality (Supplementary Table 5). All variables with a *P*-value less than 0.001 were selected as predictors for the model (Supplementary Table 6). In addition, LASSO regression was also used for variable screening, the results of which are displayed in (Supplementary Fig. 3). When λ takes the value of 0.018570, the lasso regression will output 12 variables; when λ takes the value of 0.015390, the lasso regression will output 15 variables (Supplementary Table 7). As can be seen from table, the variables screened by the lasso regression are approximately the same as those screened by the logistic regression in this study. All predictors will be incorporated into the logistic regression model a second time, and the resulting β values will be multiplied by 10 to calculate a score for each variable used to develop the model (Supplementary Table 6).

### Logistic regression model prediction of in-hospital all-cause mortality

**Fig. 2:**
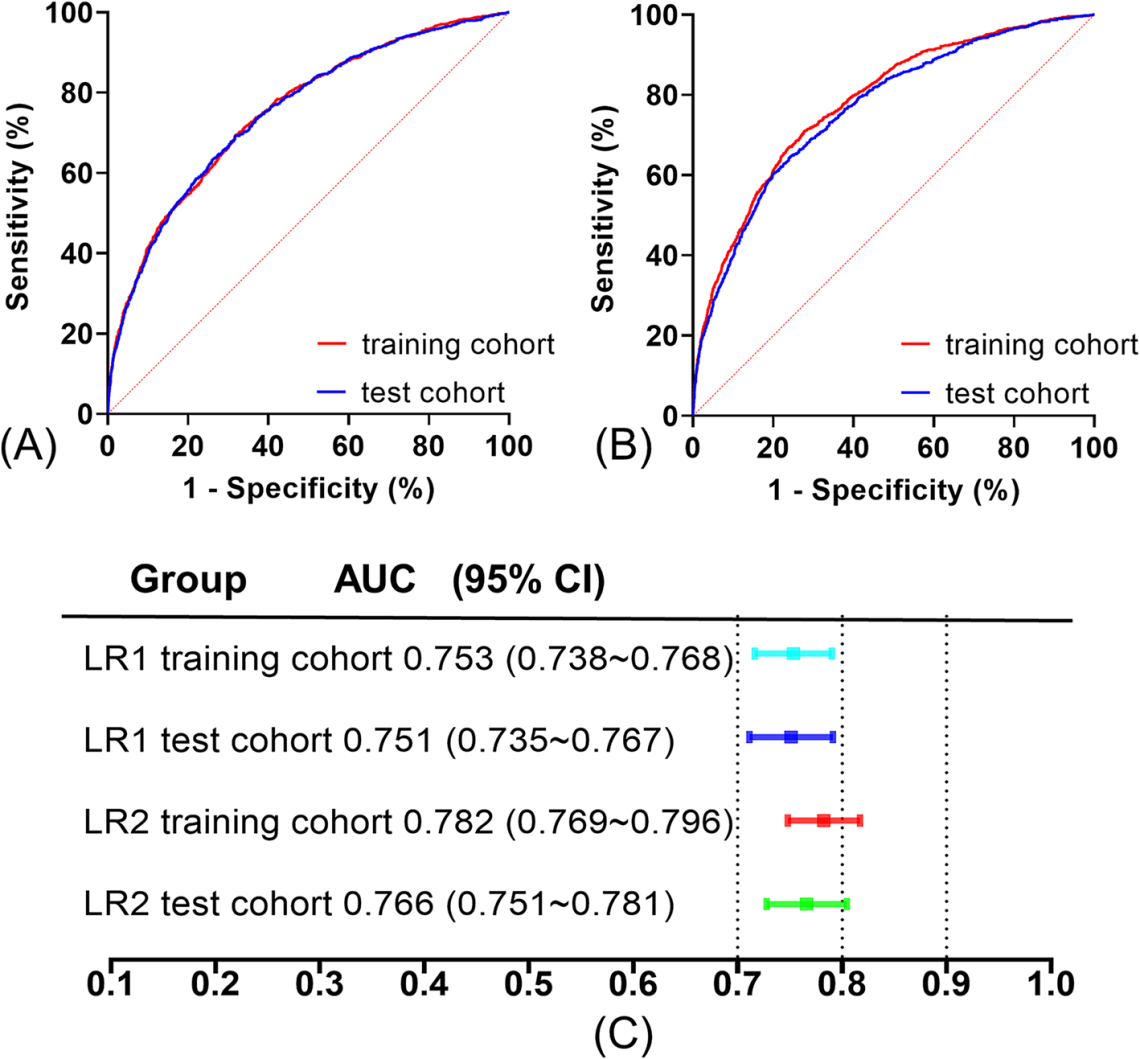
ROC curves for LR1 and LR2 model in training and test cohorts. **A** ROC curves for LR1 model in training and test cohorts; **B** ROC curves for LR2 model in training and test cohorts; **C** AUC and 95% confidence interval for LR1 and LR2 models in training and test cohorts

The receiver operating characteristic curve (ROC) of the LR1 model was plotted in the training cohort (Fig. 2A), and the area under the receiver operating characteristic curve (AUC) was calculated to be 0.753 (95% CI: 0.738∼0.768) (Fig. 2C). The AUC for the test cohort was 0.751 (95% CI: 0.735∼0.767) (Fig. 2C).

**Fig. 3:**
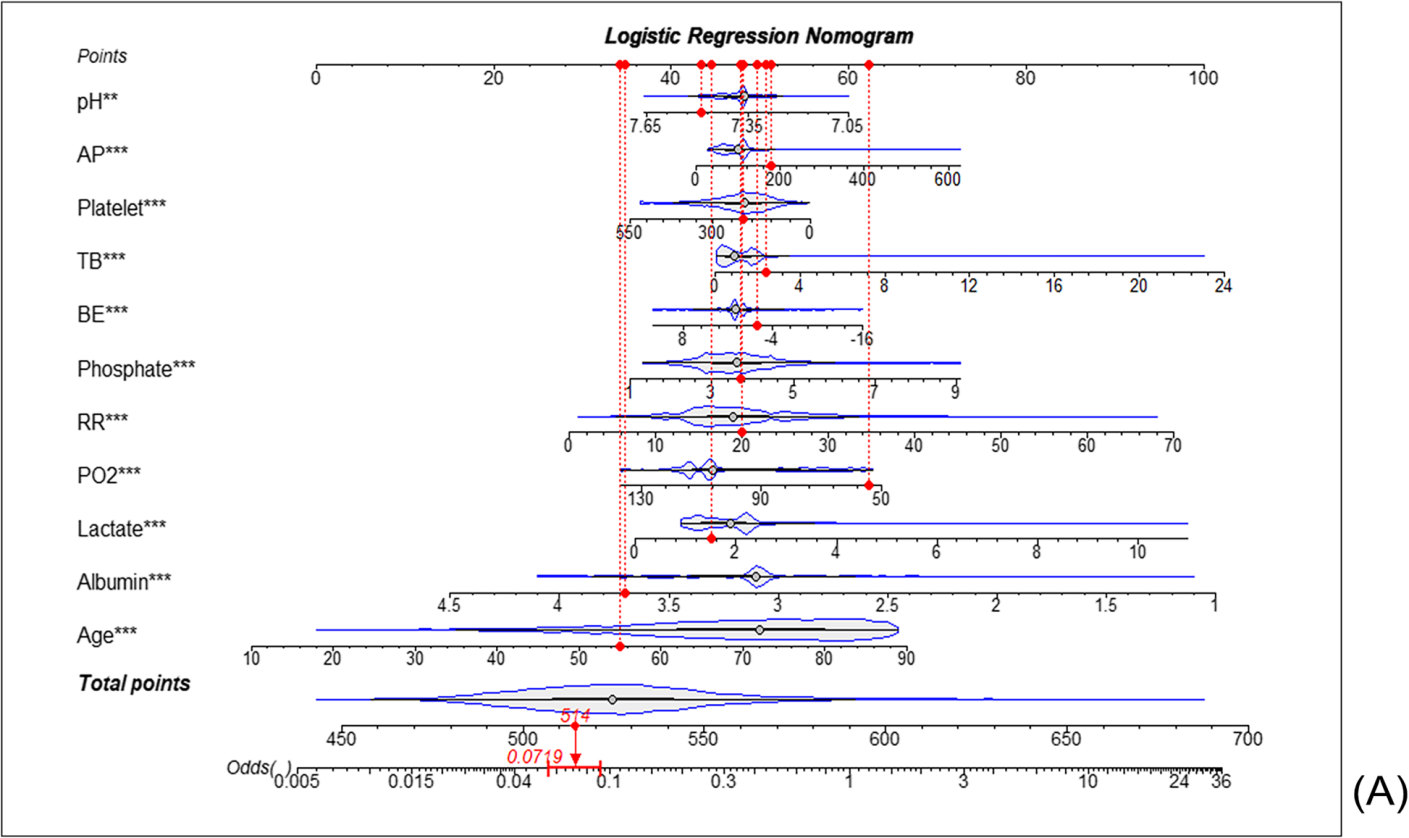
Nomogram developed to predict in-hospital all-cause mortality. Nomogram for in-hospital deaths in heart failure patients. First row: point allocation of variables; second to twelfth rows: eleven predictors; thirteenth row: total number of points for eleven predictors

The ROC curves of the LR2 model in the training and test cohorts were also plotted (Fig. 2B). The AUCs of the training and test cohorts were 0.782 (95% CI: 0.769–0.796) and 0.766 (95% CI: 0.751–0.781), respectively (Fig. 2C). Meanwhile, the 15 variables screened by the lasso regression were also used to develop the Nomogram model, which had areas under the AUC of 0.7816 (0.7677 ~ 0.7955) (*P* < 0.001, Supplementary Fig. 4A) and 0.7642 (0.7487 ~ 0.7798) (*P* < 0.001, Supplementary Fig. 4B) for the training and validation sets, respectively. The feature development model screened by lasso regression did not have a significant advantage over logistic regression. Finally, to make it easier to assess the risk of in-hospital death in patients, these variables used to model LR1 were also used to create a Nomogram for estimating the probability of in-hospital all-cause mortality (Fig. 3).

### Calibration and clinical utility of logistic regression

**Fig. 4:**
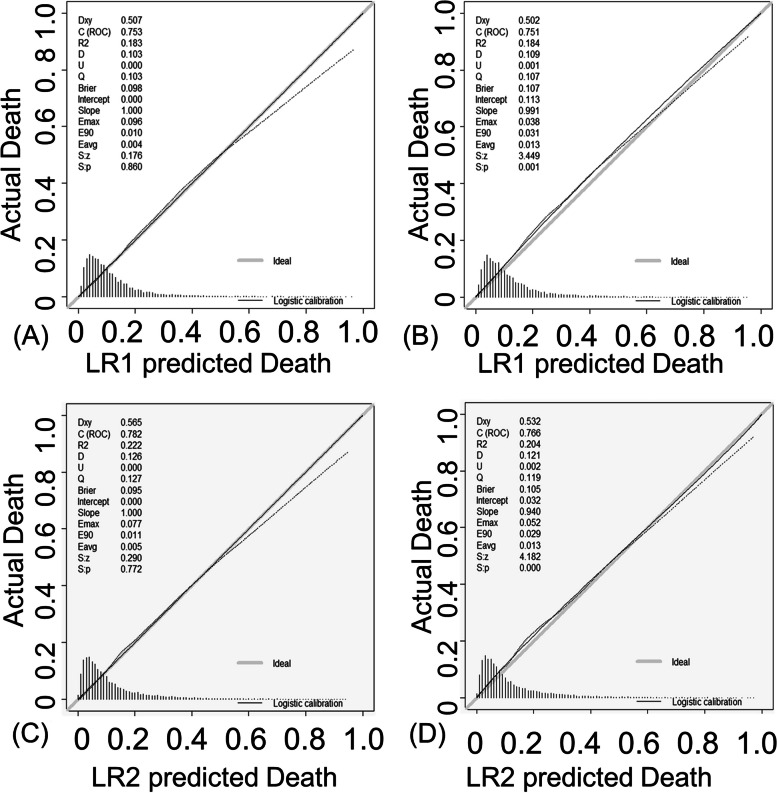
Calibration curves for LR1 and LR2 predicting in-hospital all-cause mortality in the training cohort and test cohort. **A** Calibration curves for the LR1 model predicting in-hospital all-cause mortality in the training cohort. **B** Calibration curves for the LR1 model predicting in-hospital all-cause mortality in the test cohort. **C** Calibration curves for the LR2 model predicting in-hospital all-cause mortality in the training cohort. **D** Calibration curves for the LR2 model predicting in-hospital all-cause mortality in the test cohort

For the LR1 model of in-hospital all-cause mortality, the calibration curves for the training and validation cohorts are shown in Fig. 4A and B. For the LR2 model of in-hospital all-cause mortality, the calibration curves for the training and validation cohorts are shown in Fig. 4C and D. The calibration curves show good agreement between the predicted and observed probabilities of in-hospital death in both the training and validation cohorts.

**Fig. 5:**
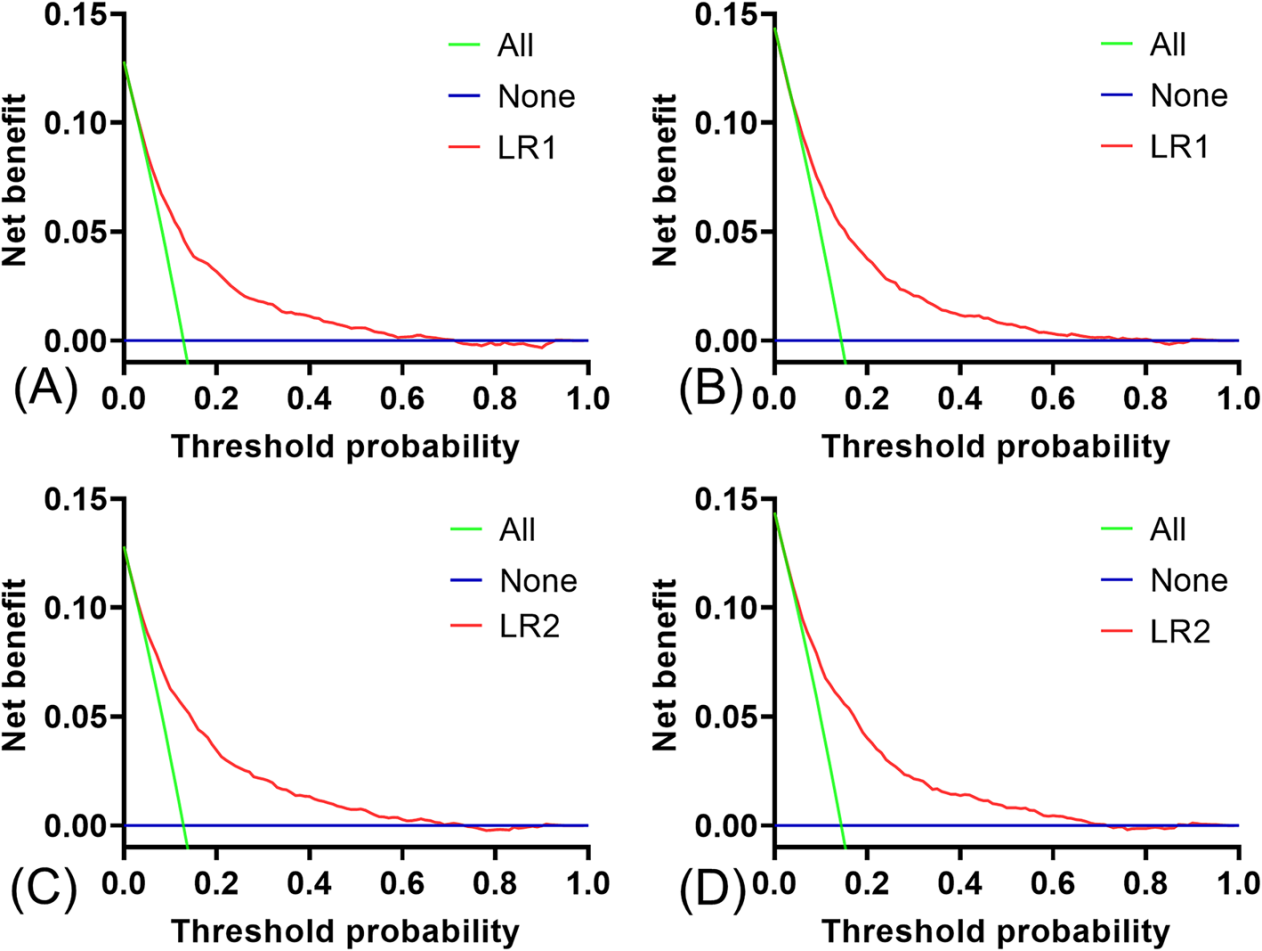
Decision curve analysis of the model. x-axis represents the threshold probability of in-hospital death and y-axis represents the net benefit. **A** DCA curves for the LR1 model predicting in-hospital all-cause mortality in the training cohort. **B** DCA curves of the LR1 model predicting in-hospital all-cause mortality in the test cohort. **C** Calibration curves for the LR2 model predicting in-hospital all-cause mortality in the training cohort. **D** Calibration curves for the LR2 model predicting in-hospital all-cause mortality in the test cohort

At the same time, the decision curve analysis also shows that the net benefit of all models exceeds that of the reference model over the entire threshold range (Fig. 5), suggesting that predictions based on LR models will more accurately identify high-risk patients and consider the pros and cons of early intervention.

### Comparison of risk predicted by LR1 model and LR2 model

**Fig. 6:**
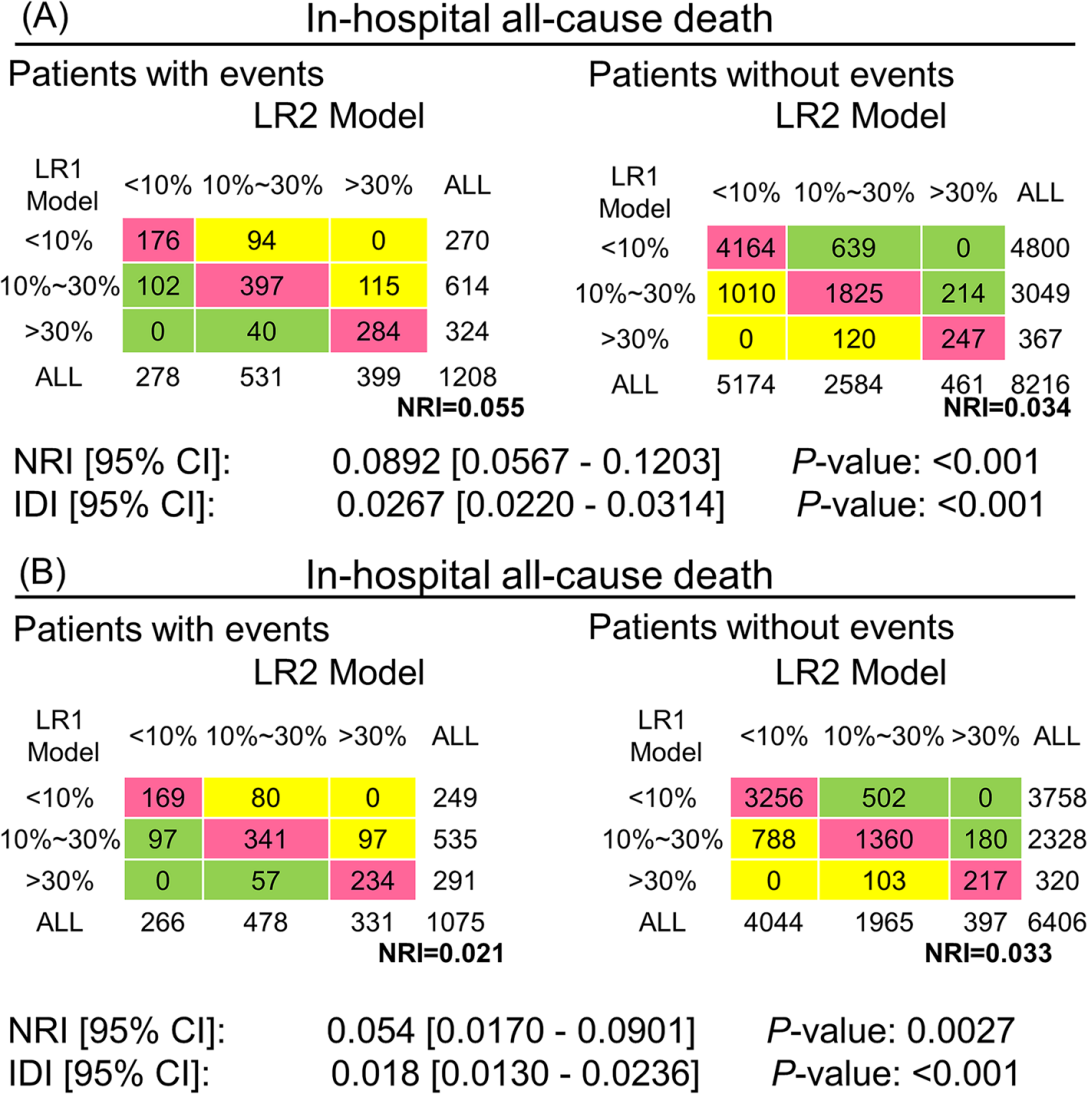
Comparison of LR1 model and LR2 for predicting in-hospital all-cause mortality. **A** NRI was calculated in the training cohort. we used 10% and 30% as thresholds to define low-risk (< 10%), intermediate-risk (10–30%), and high-risk (> 30%) patients. the IDI is also listed above. **B** Calculation of NRI and IDI in the test cohort

In the training cohort, we calculated the model-assessed risk for each patient for the LR1 and LR2 models separately, and then compared the risk estimated by the LR1 model with the risk estimated by LR2. First, the NRI was calculated in the training cohort. Referring to reported studies [18, 33], we used 10% and 30% as thresholds to define risk classes for low-risk (< 10%), intermediate risk (10%-30%), and high-risk (> 30%) patients, and compared with the LR1 model (< 10% for low risk, 10%-30% for intermediate risk, and 30% for highest risk), the LR2 model predicted an NRI of 8.92% for in-hospital all-cause mortality (Fig. 6A). Of the 1208 patients who experienced in-hospital deaths, 209 were correctly reclassified to a higher risk category by the LR2 model. On the other hand, 142 of the 1208 patients were incorrectly reclassified to a lower risk category by the LR1 model. In addition, in the test cohort, the LR2 model predicted an NRI of 5.4% for in-hospital all-cause mortality compared to the LR1 model (Fig. 6B). Of the 1075 patients with incident in-hospital mortality, 177 patients were correctly reclassified by the LR2 model into the high-risk category. On the other hand, 154 patients out of 1075 were incorrectly reclassified to the low-risk category by LR1. The IDI is also shown in (Fig. 6). The IDI calculated by the LR2 model compared to LR1 was 2.67% (*P* < 0.001) and 1.8% (*P* < 0.001) on the training and test cohort, respectively. the IDI shows the improved accuracy generated by the LR2 model. These results suggest that the LR2 model can significantly improve the prediction of HF patients compared to the LR1 model.

### Online risk assessment system for logistic regression model

**Fig. 7:**
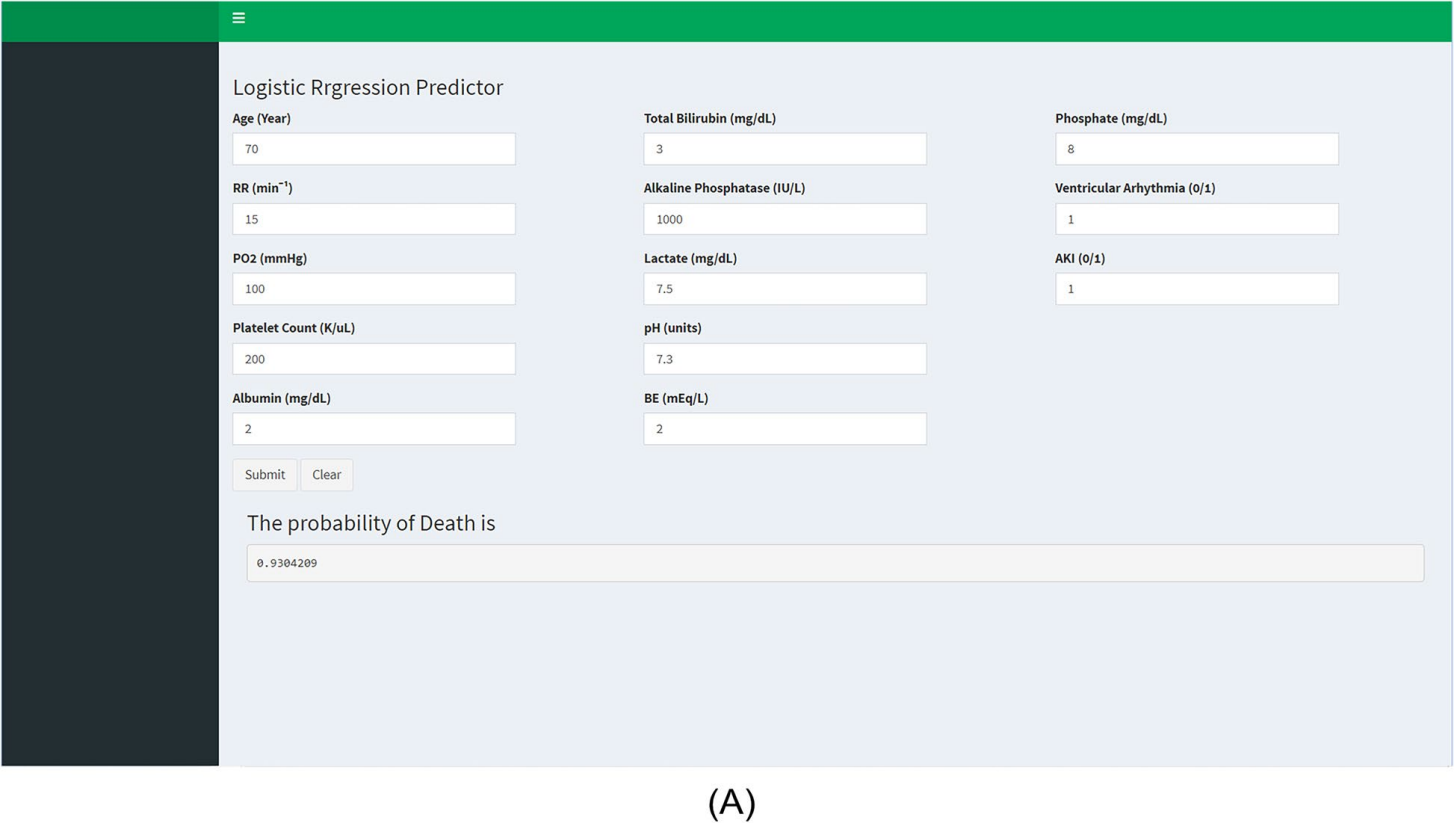
An example of an application to predict the risk of in-hospital all-cause mortality in HF patients

Ultimately, based on the predictors included in the LR2 model, 1 online application program (Fig. 7) was developed to assess the risk of in-hospital death in HF patients. The probability of in-hospital death can be calculated for each patient after admission and used to alert clinicians and identify high-risk patients as early as possible.

## Discussion

The main findings of the current study are as follows: 1) The in-hospital mortality rate of HF patients in the MIMIC database was 13.5%; 2) A total of Nomogram models was used to assess the risk of in-hospital all-cause mortality in HF patients, and we found that Nomogram had good predictive efficacy in the early assessment of the risk of in-hospital all-cause mortality in HF patients. We found that Nomogram had good predictive efficacy for early assessment of all-cause mortality risk in hospitals in HF patients, with an AUC of 0.782 in the training cohort and 0.766 in the test cohort of the LR2 model; 3) We found that age, albumin, sodium, bicarbonate, lactate, magnesium, phosphate, platelets, AG, T-CO_2_, MCV, HR, PaO2, AP, BE, RBC, RR, TB, WBC, pH, AKI and VA are independent factors influencing in-hospital mortality in HF patients.

The risk of in-hospital all-cause mortality in HF patients in previous studies was 2.86% to 14.5% [34–38]. In contrast, the in-hospital all-cause mortality of heart failure patients in this study was high. The possible reasons for this are as follows: (A) first, the median age of both MIMIC-III and MIMIC-IV was high (> 65 years) in both groups, suggesting that our study population may have more underlying disease and also the functional state of body organs is poor [39]; (B) secondly, HF as an end-stage outcome of cardiac disease is characterized by a very poor prognosis, and the mortality rate of our study population is naturally higher than that of HF patients in general wards because they are from intensive care units [40, 41]. Therefore, early identification is very important. It helps clinicians to take preventive measures in advance.

In this study, we found markers that are not specific to heart disease but are good predictors of patient prognosis. Bicarbonate was most often elevated in patients with more severe HF [42], warning of a marker of severe HF. The study have noted that serum magnesium levels less than or equal to 2 mEq/L were associated with increased cardiovascular mortality [43]. However, there have also been systematic reviews and meta-analyses showing that elevated blood magnesium is associated with an increased risk of cardiovascular (CV) mortality and all-cause mortality [44, 45]. The study of Guo W and Nakano H suggested that abnormalities in BE increase the risk of all-cause mortality [46, 47]. Unexpectedly, elevated serum phosphorus is associated with increased morbidity and mortality even when renal function is normal [48, 49]. Could AP abnormalities in HF patients, which are associated with significant signs of systemic congestion and elevated right-sided filling pressures [50], provide a new marker for the diagnosis of HF? Elevated bilirubin levels were significantly associated with the risk of death in pump failure [51, 52], suggesting that clinicians should pay more attention to bilirubin levels in HF patients and may take certain therapeutic measures as early as possible. In conclusion, the results in our clinic are broadly in line with all previously reported findings.

Using the LR model, the risk probability of the derived population was categorized into < 10%, 10–30%, and > 30%, which were defined as low-, medium-, and high-risk categories, respectively. In addition, risk stratification was also presented in the external validation dataset. We documented the feasibility of the LR model to distinguish risky patients from other populations. By using the LR model, the risk probability of each patient can inform and support the clinician’s decision making. However, there were some deaths in the low-risk stratum and some survivors in the high-risk stratum. We suspect that these exceptions may be due to the different phenotypes of HF patients in the various risk strata. HF involves multiple pathophysiologic mechanisms, which may lead to clinically heterogeneous phenotypes [53]. For example, unsupervised clustering analysis based on machine learning was used to differentiate between different phenotypes of heart failure with preserved ejection fraction (HFpEF) patients [54]. Therefore, in future studies, we may use other methods for further analysis and perform experimental validation.

Peng S et al. developed a clinical prediction model for 28-day all-cause in-hospital mortality in critically ill patients with heart failure combined with hypertension using machine learning, in which Neural Network (NN) performed the best, with an AUC of 0.764 [55]. Li J et al. developed several machine learning models, and found that XGBoost, LR models performed excellently [56]. The logistic regression model was effective in improving the accuracy of risk stratification for in-hospital mortality in patients with HF. However, the sample size of this study was relatively small and included many variables, which is not conducive to clinical generalization. With the development of concepts such as real-world research and precision therapy, there is an increasing demand for medical big data processing by researchers. Therefore, we tried to explore a predictive model for the risk of in-hospital death in heart failure with a larger sample size and more robustness from another study.

We tried to develop a new model rather than validate the original model. The reason for this is that the variables included in previously developed models are not fully accessible. For example, the H2FPEF and HFA-PEFF scores [57] developed by Ouwerkerk W et al. for the diagnosis of ejection fraction preserved heart failure, and the more commonly used Meta-analysis Global Group in Chronic Heart Failure (MAGGIC) score [58]. Both performed well, but the former contains cardiac ultrasound data, and the latter contains BMI, NYHA classification, and other metrics not available from the MIMIC database. We had to abandon the validation of the developed model in favor of developing a new one.

This study used a high quality, large sample size database, MIMIC. there are several advantages to using the database. First, it is one of the few critical care databases that is freely accessible. Second, the dataset spans more than a decade and contains a wealth of detailed information about patient care. Third, once data use agreements are accepted, there are no restrictions on analysis by researchers, enabling clinical research and education around the world. Finally, data can be downloaded from multiple sources [22].

There are several limitations in the current study. Firstly, although the internal validation of the model yielded the best discrimination and excellent calibration, the data came from public databases. Therefore, the generalizability of the column plot still needs to be externally validated using other medical centers. Further training in prospective studies could significantly improve the predictive performance and stability of the column plot; Second, although the column chart has been widely used in clinical practice to assist in medical decision making, we would like to further simplify the model and expand its usage scenarios. Finally, the model can be significantly improved by incorporating imaging data, such as cardiac ultrasound, electrocardiogram, and other parameters, or circulating biomarkers that are more predictive.

## Conclusion

A new risk prediction tool and an online risk assessment system were developed to predict mortality in HF patients, which performed well and might be used to guide clinical practice.

### Supplementary Information

**Additional file 1: Supplementary Figure 1.** Proportion missing before filling for all continuous variables in the MIMIC III database. **Supplementary Figure 2.** Proportion missing before filling for all continuous variables in the MIMIC IV database. **Supplementary Figure 3.** LASSO regression results are shown; (A), LASSO coefficient path; (B), LASSO regularization path. **Supplementary Figure 4.** LASSO regression screened 15 variables to develop a Nomogram model; (A), training cohort ROC curves, and AUC with 95% confidence intervals; (B), test cohort ROC curves, and AUC with 95% confidence intervals.

**Additional file 2: Supplementary Table 1.** Statistical table of missing values for all continuous variables. **Supplementary Table 2.** Baseline table of subjects for the entire cohort. **Supplementary Table 3.** Baseline characteristics of patients in the MIMIC III cohort and MIMIC IV cohort. **Supplementary Table 4.** Baseline information for patients in the MIMIC IV cohort. **Supplementary Table 5.** Univariate logistic regression analysis results and multivariate logistic regression analysis results of all variables. **Supplementary Table 6.** Model Variable Score Table. **Supplementary Table 7.** Feature Screening.

## Data Availability

The datasets presented in this study can be found in online repositories. The names of the repository/repositories and accession number(s) can be found below: https://physionet.org/content/mimiciii/1.4/ and https://physionet.org/content/mimiciv/2.2/. In-hospital AKI diagnoses can also be accessed directly through the officially provided view codes https://github.com/MIT-LCP/mimic-code/.
