# Supplementary material for: Development and validation of a novel combinatorial nomogram model to predict in-hospital deaths in heart failure patients
Source: BMC Cardiovasc Disord. 2024 Jan 3;24:16. doi: 10.1186/s12872-023-03683-0 (PMC10765573; doi:10.1186/s12872-023-03683-0)

**Supplementary Figure 1**: Proportion missing before filling for all continuous variables in the MIMIC Ⅲ database


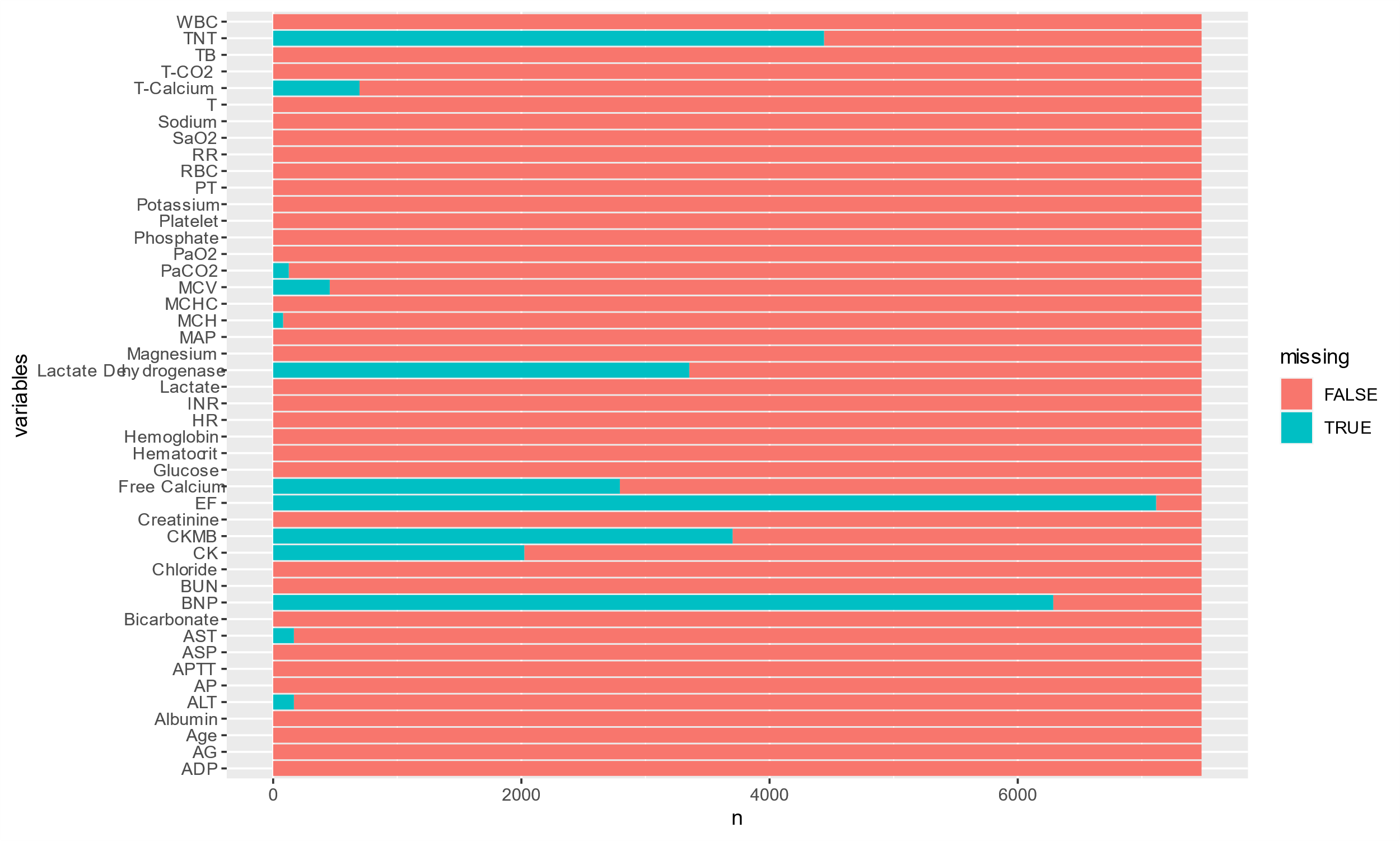


**Supplementary Figure 2**: Proportion missing before filling for all continuous variables in the MIMIC Ⅳ database


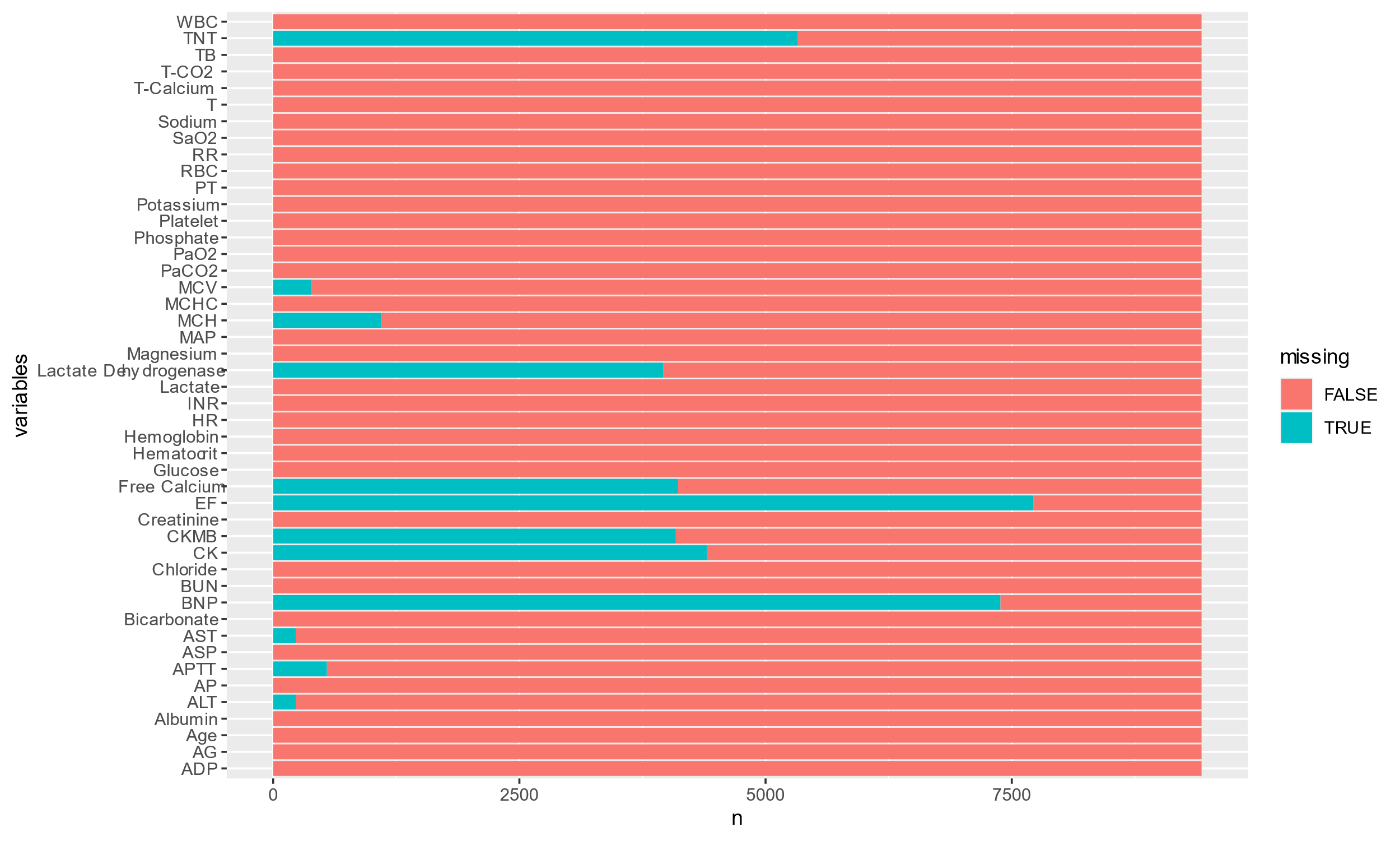


**Supplementary Figure 3**: LASSO regression results are shown; (A), LASSO coefficient path; (B), LASSO regularization path.


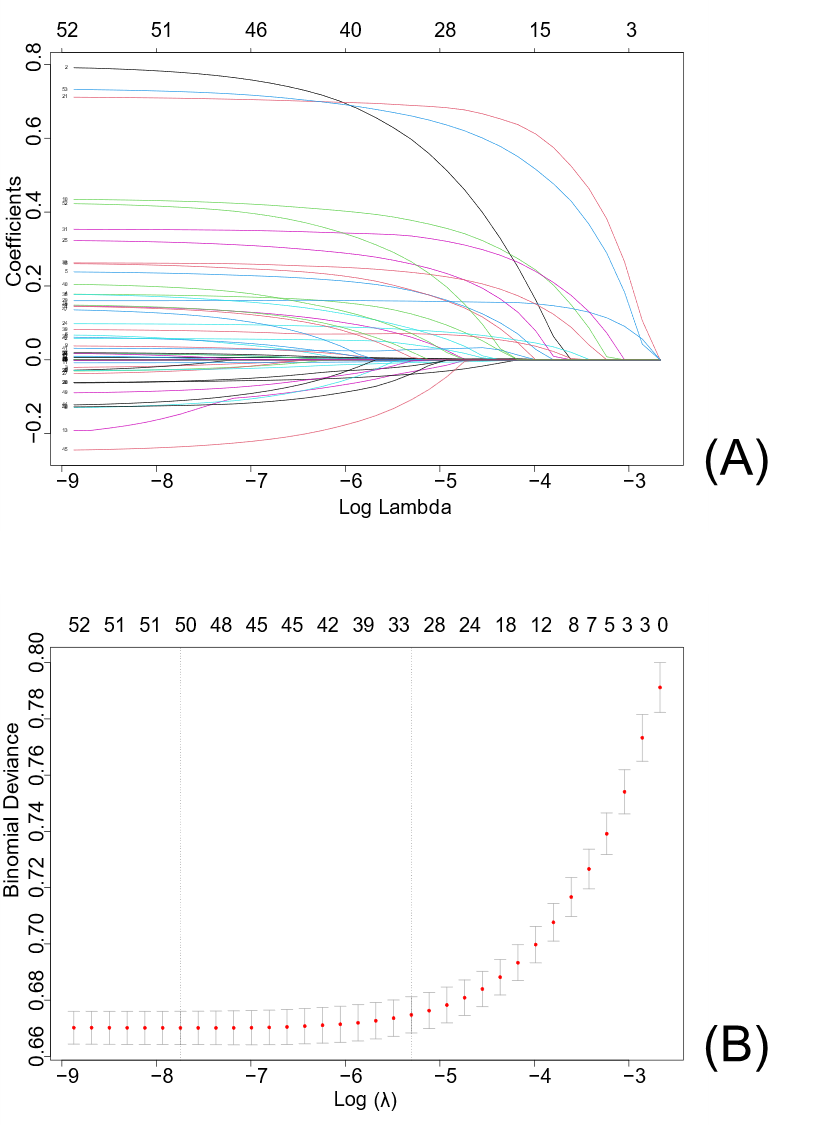


**Supplementary Figure 4**: LASSO regression screened 15 variables to develop a column-line graphical model; (A), training cohort ROC curves, and AUC with 95% confidence intervals; (B), test cohort ROC curves, and AUC with 95% confidence intervals.


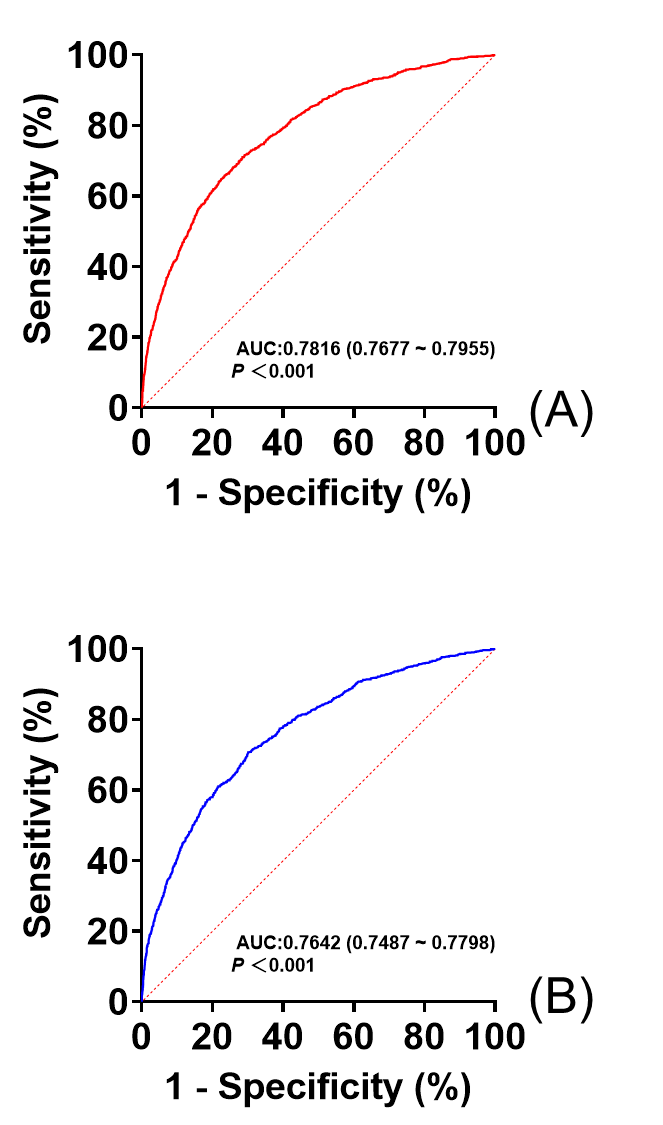

Supplement: Supplementary file 1 — Additional file 1: Supplementary Figure 1. Proportion missing before filling for all continuous variables in the MIMIC III database. Supplementary Figure 2. Proportion missing before filling for all continuous variables in the MIMIC IV database. Supplementary Figure 3. LASSO regression results are shown; (A), LASSO coefficient path; (B), LASSO regularization path. Supplementary Figure 4. LASSO regression screened 15 variables to develop a Nomogram model; (A), training cohort ROC curves, and AUC with 95% confidence intervals; (B), test cohort ROC curves, and AUC with 95% confidence intervals. [file 12872_2023_3683_MOESM1_ESM.docx]
