# Supplementary material for: Development and validation of a novel combinatorial nomogram model to predict in-hospital deaths in heart failure patients
Source: BMC Cardiovasc Disord. 2024 Jan 3;24:16. doi: 10.1186/s12872-023-03683-0 (PMC10765573; doi:10.1186/s12872-023-03683-0)
Supplement: Supplementary file 2 — Additional file 2: Supplementary Table 1. Statistical table of missing values for all continuous variables. Supplementary Table 2. Baseline table of subjects for the entire cohort. Supplementary Table 3. Baseline characteristics of patients in the MIMIC III cohort and MIMIC IV cohort. Supplementary Table 4. Baseline information for patients in the MIMIC IV cohort. Supplementary Table 5. Univariate logistic regression analysis results and multivariate logistic regression analysis results of all variables. Supplementary Table 6. Model Variable Score Table. Supplementary Table 7. Feature Screening. [file 12872_2023_3683_MOESM2_ESM.docx]

Supplementary table 1: Statistical table of missing values for all continuous variables

| **Univariate statistics** | | | |
| --- | --- | --- | --- |
|  | Number of cases | Missing | |
|  |  | Counting | Percentage |
| Age | 16908 | 0 | 0.00 |
| Heart rata | 16908 | 0 | 0.00 |
| Respiratory rate | 16908 | 0 | 0.00 |
| Systolic blood pressure | 16908 | 0 | 0.00 |
| Diastolic blood pressure | 16908 | 0 | 0.00 |
| Mean arterial pressure | 16908 | 0 | 0.00 |
| Temperature | 16908 | 0 | 0.00 |
| Arterial oxygen saturation | 16908 | 0 | 0.00 |
| Arterial partial pressure of carbon-dioxide | 16782 | 126 | 0.75 |
| Arterial oxygen saturation | 16908 | 0 | 0.00 |
| Red blood cell | 16908 | 0 | 0.00 |
| White blood cell | 16908 | 0 | 0.00 |
| Platelet | 16908 | 0 | 0.00 |
| Hemoglobin | 16908 | 0 | 0.00 |
| Hematocrit | 16908 | 0 | 0.00 |
| Mean corpuscular volume | 16063 | 845 | 5.00 |
| Mean corpuscular hemoglobin | 15732 | 1176 | 6.96 |
| Mean corpuscular hemoglobin concentration | 16908 | 0 | 0.00 |
| Albumin | 16908 | 0 | 0.00 |
| Alanine transaminase | 16512 | 396 | 2.34 |
| Aspertate Aminotransferase | 16512 | 396 | 2.34 |
| Total Bilirubin | 16908 | 0 | 0.00 |
| Alkaline phosphatase | 16908 | 0 | 0.00 |
| Blood urea nitrogen | 16908 | 0 | 0.00 |
| Creatinine | 16908 | 0 | 0.00 |
| Glucose | 16908 | 0 | 0.00 |
| Creatine kinase | 10479 | 6429 | 38.02 |
| Creatine kinase-MB | 9117 | 7791 | 46.08 |
| Troponin-T | 7145 | 9763 | 57.74 |
| lactic dehydrogenase | 9593 | 7315 | 43.26 |
| Lactate | 16908 | 0 | 0.00 |
| Total carbon dioxide | 16908 | 0 | 0.00 |
| Potential of hydrogen | 16908 | 0 | 0.00 |
| Anion gap | 16908 | 0 | 0.00 |
| Base excess | 16908 | 0 | 0.00 |
| Bicarbonate | 16908 | 0 | 0.00 |
| Potassium | 16908 | 0 | 0.00 |
| Sodium | 16908 | 0 | 0.00 |
| Chloride | 16908 | 0 | 0.00 |
| Free Calcium | 10002 | 6906 | 40.84 |
| Ejection Fraction | 2074 | 14834 | 87.7 |
| Brain Natriuretic Peptide | 3238 | 13670 | 80.8 |
| Total Calcium | 16212 | 696 | 4.12 |
| Magnesium | 16908 | 0 | 0.00 |
| Phosphate | 16908 | 0 | 0.00 |
| International Normalized Ratio | 16908 | 0 | 0.00 |
| Activated partial prothrombin time | 16363 | 545 | 3.22 |
| Prothrombin time | 16908 | 0 | 0.00 |

Supplementary table 2. Baseline table of subjects for the entire cohort

|  | Overall (n=16908) | Dead (n=2283) | Survival (n=14625) | *P-value* |
| --- | --- | --- | --- | --- |
| **Demographic** |  |  |  |  |
| Age (Year) | 70.04 (13.05) | 73.09 (12.34) | 69.56 (13.10) | <0.001 |
| Male (n%) | 9092 (53.8) | 1204 (52.7) | 7888 (53.9) | 0.296 |
| **Vital signs** |  |  |  |  |
| HR (minˉ¹) | 89.22 (20.13) | 93.32 (22.22) | 88.58 (19.71) | <0.001 |
| RR (minˉ¹) | 19.60 (6.30) | 21.61 (6.85) | 19.29 (6.16) | <0.001 |
| SBP (mmHg) | 122.01 (24.16) | 117.34 (26.33) | 122.74 (23.72) | <0.001 |
| DBP (mmHg) | 65.73 (16.81) | 64.35 (17.73) | 65.95 (16.66) | <0.001 |
| MAP (mmHg) | 84.49 (16.77) | 82.01 (17.89) | 84.88 (16.55) | <0.001 |
| T (°C) | 36.60 [36.20, 37.10] | 36.70 (1.17) | 36.67 (0.82) | 0.108 |
| **Laboratory results** |  |  |  |  |
| RBC (m/uL) | 3.80 (0.76) | 3.69 (0.78) | 3.82 (0.76) | <0.001 |
| WBC (k/uL) | 10.10 [7.30, 13.80] | 11.20 [7.80, 15.90] | 9.90 [7.30, 13.50] | <0.001 |
| Platelet (k/uL) | 209.00 [157.00, 265.00] | 199.00 [135.00, 264.00] | 210.00 [160.00, 265.00] | <0.001 |
| Hemoglobin (g/dL) | 11.30 (2.24) | 10.96 (2.18) | 11.35 (2.24) | <0.001 |
| Hematocrit (%) | 34.31 (6.52) | 33.59 (6.54) | 34.43 (6.51) | <0.001 |
| MCV (fL) | 90.42 (6.85) | 91.48 (7.45) | 90.25 (6.73) | <0.001 |
| MCH (pg) | 29.82 (2.71) | 29.91 (2.91) | 29.81 (2.68) | 0.119 |
| MCHC (%) | 32.96 (1.67) | 32.64 (1.72) | 33.01 (1.66) | <0.001 |
| Albumin (mg/dL) | 3.15 (0.45) | 2.95 (0.55) | 3.18 (0.42) | <0.001 |
| ALT (IU/L) | 38.00 [19.00, 88.00] | 36.00 [19.00, 88.00] | 39.00 [18.00, 88.00] | 0.448 |
| AST (IU/L) | 50.00 [25.00, 118.00] | 57.00 [28.00, 132.00] | 49.00 [25.00, 117.00] | <0.001 |
| TB (mg/dL) | 0.90 [0.50, 1.70] | 0.90 [0.50, 1.80] | 0.90 [0.50, 1.70] | 0.001 |
| AP (IU/L) | 103.00 [70.00, 130.00] | 107.00 [73.00, 130.00] | 103.00 [69.00, 130.00] | <0.001 |
| BUN (mg/dL) | 23.00 [16.00, 35.00] | 30.00 [19.00, 46.00] | 22.00 [16.00, 33.00] | <0.001 |
| Creatinine (mg/dL) | 1.30 [1.10, 1.50] | 1.40 [1.20, 1.80] | 1.30 [1.10, 1.50] | <0.001 |
| Glucose (mg/dL) | 129.00 [104.00, 169.00] | 133.00 [104.00, 179.00] | 128.00 [104.00, 167.00] | 0.045 |
| Lactate (mg/dL) | 2.00 [1.40, 2.50] | 2.20 [1.50, 3.40] | 2.00 [1.30, 2.50] | <0.001 |
| T-CO2 (mEq/L) | 25.00 [24.00, 28.00] | 25.00 [20.00, 28.00] | 25.00 [25.00, 28.00] | <0.001 |
| pH (units) | 7.37 (0.08) | 7.34 (0.11) | 7.38 (0.08) | <0.001 |
| PO_2_ (mmHg) | 100.02 (23.34) | 92.61 (24.14) | 101.18 (23.00) | <0.001 |
| PCO_2_ (mmHg) | 43.08 (10.23) | 43.04 (13.09) | 43.08 (9.71) | 0.844 |
| SaO_2_ (%) | 95.48 (6.40) | 94.47 (7.34) | 95.64 (6.23) | <0.001 |
| AG (mEq/L) | 15.02 (3.74) | 16.20 (4.30) | 14.83 (3.61) | <0.001 |
| BE (mEq/L) | 0.00 [-1.00, 2.00] | 0.00 [-5.00, 1.00] | 0.00 [0.00, 2.00] | <0.001 |
| Bicarbonate (mg/dL) | 24.52 (5.09) | 23.28 (5.72) | 24.72 (4.96) | <0.001 |
| Potassium (mEq/L) | 4.30 (0.77) | 4.41 (0.87) | 4.28 (0.75) | <0.001 |
| Sodium (mEq/L) | 138.05 (4.72) | 137.57 (5.63) | 138.12 (4.56) | <0.001 |
| Chloride (mEq/L) | 102.24 (6.12) | 101.70 (6.68) | 102.33 (6.02) | <0.001 |
| T-Calcium (mEq/L) | 8.56 (0.78) | 8.40 (0.91) | 8.58 (0.75) | <0.001 |
| Magnesium (mg/dL) | 1.99 (0.38) | 2.01 (0.40) | 1.99 (0.38) | 0.002 |
| Phosphate (mg/dL) | 3.60 [3.00, 4.20] | 3.80 [3.10, 4.70] | 3.60 [3.00, 4.10] | <0.001 |
| INR | 1.30 [1.10, 1.60] | 1.40 [1.20, 1.85] | 1.30 [1.10, 1.60] | <0.001 |
| APTT (s) | 31.00 [27.10, 37.60] | 32.50 [27.30, 39.20] | 30.90 [27.10, 37.40] | 0.003 |
| PT (s) | 14.20 [12.80, 17.20] | 15.10 [13.20, 19.60] | 14.10 [12.70, 16.90] | <0.001 |
| **Comorbidities (n%)** |  |  |  |  |
| AF | 5711 (33.8) | 820 (35.9) | 4891 (33.4) | 0.021 |
| DM | 3322 (19.6) | 392 (17.2) | 2930 (20.0) | 0.002 |
| Hypertension | 6640 (39.3) | 765 (33.5) | 5875 (40.2) | <0.001 |
| p-MI | 1426 (8.4) | 152 (6.7) | 1274 (8.7) | 0.001 |
| VA | 1548 (9.2) | 296 (13.0) | 1252 (8.6) | <0.001 |
| AKI | 6158 (36.4) | 1331 (58.3) | 4827 (33.0) | <0.001 |
| CLD | 4986 (29.5) | 707 (31.0) | 4279 (29.3) | 0.101 |
| MT | 1069 (6.3) | 161 (7.1) | 908 (6.2) | 0.135 |
| CKD | 12908 (76.3) | 1755 (76.9) | 11153 (76.3) | 0.539 |
| Anemia | 7232 (42.8) | 989 (43.3) | 6243 (42.7) | 0.585 |

HR, Heart rata; RR, Respiratory rate; SBP, Systolic blood pressure; DBP, Diastolic blood pressure; MAP, Mean arterial pressure; T, Temperature; RBC, Red blood cell; WBC, White blood cell; MCV, Mean corpuscular volume; MCH, Mean corpuscular hemoglobin; MCHC, Mean corpuscular hemoglobin concentration; ALT, Alanine transaminase; AST, aspartate aminotransferase; TB, Total Bilirubin; AP, Alkaline phosphatase; BUN, blood urea nitrogen; T-CO_2_, Total carbon dioxide; pH, potential of hydrogen; PaO_2_, arterial partial pressure of oxygen; PaCO_2_, arterial partial pressure of carbon-dioxide; SaO_2_, arterial oxygen saturation; AG, anion gap; BE, base excess; INR, International Normalized Ratio; PT, prothrombin time; APTT, activated partial prothrombin time; AF, atrial fibrillation; DM, Diabetes Mellitus; p-MI, previous myocardial infarction; VA, ventricular arrhythmias; AKI, acute kidney injury. CLD, chronic lung disease; MT, malignant tumor; CKD, chronic kidney diseases.

Supplementary table 3. Baseline characteristics of patients in the MIMIC III cohort and MIMIC IV cohort.

|  | MIMIC Ⅲ (n=7481) | MIMIC Ⅳ (n=9427) | *P*-value |
| --- | --- | --- | --- |
| **Demographic** |  |  |  |
| Age (year) | 70.37 (13.03) | 69.78 (13.06) | 0.003 |
| Male | 4003 (53.5) | 5089 (54.0) | 0.549 |
| **Vital signs** |  |  |  |
| HR (bpm) | 89.44 (19.79) | 89.04 (20.39) | 0.196 |
| RR (bpm) | 19.25 (6.31) | 19.88 (6.28) | <0.001 |
| SBP (mmHg) | 121.85 (24.31) | 122.15 (24.05) | 0.427 |
| DBP (mmHg) | 63.49 (15.08) | 67.51 (17.87) | <0.001 |
| MAP (mmHg) | 82.95 (15.78) | 85.72 (17.42) | <0.001 |
| T (°C) | 36.60 [36.00, 37.10] | 36.70 [36.40, 37.10] | <0.001 |
| **Laboratory results** |  |  |  |
| RBC (m/uL) | 3.88 (0.75) | 3.73 (0.77) | <0.001 |
| WBC (k/uL) | 10.40 [7.50, 14.10] | 9.80 [7.20, 13.50] | <0.001 |
| Platelet (k/uL) | 218.00 [167.00, 272.00] | 201.00 [151.00, 259.00] | <0.001 |
| Hemoglobin (g/dL) | 11.59 (2.22) | 11.07 (2.22) | <0.001 |
| Hematocrit (%) | 34.72 (6.40) | 33.99 (6.59) | <0.001 |
| MCV (fL) | 89.73 (6.75) | 90.96 (6.87) | <0.001 |
| MCH (pg) | 30.00 (2.66) | 29.68 (2.75) | <0.001 |
| MCHC (%) | 33.45 (1.57) | 32.57 (1.64) | <0.001 |
| Albumin (mg/dL) | 3.13 (0.45) | 3.17 (0.45) | <0.001 |
| ALT (IU/L) | 37.00 [19.00, 88.00] | 38.00 [18.00, 96.00] | <0.001 |
| AST (IU/L) | 49.00 [25.00, 117.00] | 49.00 [25.00, 137.00] | <0.001 |
| TB (mg/dL) | 0.90 [0.50, 1.90] | 0.90 [0.50, 1.70] | <0.001 |
| AP (IU/L) | 108.00 [72.00, 130.00] | 100.00 [69.00, 116.00] | <0.001 |
| BUN (mg/dL) | 23.00 [17.00, 35.00] | 22.00 [16.00, 35.00] | <0.001 |
| Creatinine (mg/dL) | 1.30 [1.10, 1.50] | 1.30 [1.10, 1.50] | 0.838 |
| Glucose (mg/dL) | 134.00 [107.00, 174.00] | 126.00 [101.00, 165.00] | <0.001 |
| Lactate (mg/dL) | 2.40 [1.40, 2.50] | 1.90 [1.30, 2.20] | <0.001 |
| T-CO2 (mEq/L) | 25.00 [24.00, 29.00] | 25.00 [24.00, 28.00] | 0.001 |
| pH (units) | 7.37 (0.09) | 7.37 (0.08) | 0.011 |
| PaO_2_ (mmHg) | 104.19 (25.38) | 96.72 (21.01) | <0.001 |
| PaCO_2_ (mmHg) | 43.11 (10.73) | 43.06 (9.82) | 0.749 |
| SaO_2_ (%) | 95.31 (6.68) | 95.61 (6.16) | 0.003 |
| AG (mEq/L) | 15.21 (3.76) | 14.86 (3.72) | <0.001 |
| BE (mEq/L) | 0.00 [-1.00, 2.00] | 1.00 [-1.00, 2.00] | <0.001 |
| Bicarbonate (mg/dL) | 24.68 (5.20) | 24.40 (5.01) | <0.001 |
| Potassium (mEq/L) | 4.35 (0.80) | 4.25 (0.74) | <0.001 |
| Sodium (mEq/L) | 137.94 (4.60) | 138.12 (4.82) | 0.014 |
| Chloride (mEq/L) | 102.20 (5.98) | 102.28 (6.23) | 0.422 |
| T-Calcium (mEq/L) | 8.58 (0.76) | 8.54 (0.76) | <0.001 |
| Magnesium (mg/dL) | 1.97 (0.39) | 2.00 (0.38) | <0.001 |
| Phosphate (mg/dL) | 3.60 [3.00, 4.20] | 3.60 [3.00, 4.20] | 0.092 |
| INR | 1.30 [1.10, 1.60] | 1.30 [1.10, 1.50] | 0.516 |
| APTT (s) | 30.40 [26.30, 36.80] | 32.00 [27.95, 38.30] | <0.001 |
| PT (s) | 14.20 [13.00, 16.50] | 14.20 [12.50, 18.10] | 0.022 |
| **Comorbidities (n%)** |  |  |  |
| AF | 3148 (42.1) | 2563 (27.2) | <0.001 |
| DM | 1856 (24.8) | 1466 (15.6) | <0.001 |
| Hypertension | 3628 (48.5) | 3012 (32.0) | <0.001 |
| p-MI | 676 (9.0) | 750 (8.0) | 0.013 |
| VA | 693 (9.3) | 855 (9.1) | 0.684 |
| AKI | 2728 (36.5) | 3430 (36.4) | 0.926 |
| CLD | 2670 (35.7) | 2316 (24.6) | <0.001 |
| MT | 398 (5.3) | 671 (7.1) | <0.001 |
| CKD | 5806 (77.6) | 7102 (75.3) | 0.001 |
| Anemia | 2852 (38.1) | 4380 (46.5) | <0.001 |
| **Study Endpoint** |  |  |  |
| Death in hospital | 1075 (14.4) | 1208 (12.8) | 0.004 |

HR, Heart rata; RR, Respiratory rate; SBP, Systolic blood pressure; DBP, Diastolic blood pressure; MAP, Mean arterial pressure; T, Temperature; RBC, Red blood cell; WBC, White blood cell; MCV, Mean corpuscular volume; MCH, Mean corpuscular hemoglobin; MCHC, Mean corpuscular hemoglobin concentration; ALT, Alanine transaminase; AST, aspartate aminotransferase; TB, Total Bilirubin; AP, Alkaline phosphatase; BUN, blood urea nitrogen; T-CO_2_, Total carbon dioxide; pH, potential of hydrogen; PaO_2_, arterial partial pressure of oxygen; PaCO_2_, arterial partial pressure of carbon-dioxide; SaO_2_, arterial oxygen saturation; AG, anion gap; BE, base excess; INR, International Normalized Ratio; PT, prothrombin time; APTT, activated partial prothrombin time; AF, atrial fibrillation; DM, Diabetes Mellitus; p-MI, previous myocardial infarction; VA, ventricular arrhythmias; AKI, acute kidney injury, CLD, chronic lung disease; MT, malignant tumor; CKD, chronic kidney diseases.

Supplementary table 4. Baseline information for patients in the MIMIC IV cohort

|  | **MIMIC Ⅳ(N=9427)** | | | |
| --- | --- | --- | --- | --- |
|  | **Survival (n=8219)** | | **Dead (n=1208)** | ***P-value*** |
| **Demographic** |  | |  |  |
| Age (Year) | 69.29 (13.13) | | 73.05 (12.15) | <0.001 |
| Male (n%) | 4443 (54.1) | | 646 (53.5) | 0.728 |
| **Vital signs** |  | |  |  |
| HR (minˉ¹) | 88.40 (20.01) | | 93.40 (22.37) | <0.001 |
| RR (minˉ¹) | 19.57 (6.13) | | 21.93 (6.91) | <0.001 |
| SBP (mmHg) | 122.96 (23.79) | | 116.62 (25.01) | <0.001 |
| DBP (mmHg) | 67.68 (17.67) | | 66.36 (19.20) | 0.017 |
| MAP (mmHg) | 86.11 (17.21) | | 83.11 (18.55) | <0.001 |
| T (°C) | 36.70 [36.40,37.10] | | 36.70 [36.20,37.12] | 0.06 |
| **Laboratory results** | |  |  |  |
| RBC (m/uL) | 3.75 (0.77) | | 3.60 (0.79) | <0.001 |
| WBC (k/uL) | 9.60 [7.10,13.30] | | 11.20 [7.90,16.10] | <0.001 |
| Platelet (k/uL) | 202.00 [153.00,259.00] | | 192.00 [131.00,258.25] | <0.001 |
| Hemoglobin (g/dL) | 11.13 (2.22) | | 10.64 (2.18) | <0.001 |
| Hematocrit (%) | 34.13 (6.56) | | 33.02 (6.67) | <0.001 |
| MCV (fL) | 91.08 (6.81) | | 90.83 (6.98) | 0.226 |
| MCH (pg) | 29.94 (2.56) | | 29.88 (2.62) | 0.202 |
| MCHC (%) | 32.62 (1.63) | | 32.22 (1.68) | <0.001 |
| Albumin (mg/dL) | 3.20 (0.42) | | 2.97 (0.55) | <0.001 |
| ALT (IU/L) | 39.00 [18.00, 96.00] | | 36.00 [18.00, 96.00] | 0.929 |
| AST (IU/L) | 49.00 [25.00, 137.00] | | 55.50 [28.00, 137.00] | <0.001 |
| TB (mg/dL) | 0.90 [0.50,1.70] | | 0.90 [0.50,1.70] | 0.043 |
| AP (IU/L) | 100.00 [68.00,116.00] | | 103.00 [72.00,125.25] | <0.001 |
| BUN (mg/dL) | 22.00 [16.00,33.00] | | 29.00 [19.00,45.00] | <0.001 |
| Creatinine (mg/dL) | 1.30 [1.10,1.50] | | 1.40 [1.20,1.80] | <0.001 |
| Glucose (mg/dL) | 125.00 [102.00,163.00] | | 128.50 [101.00,177.00] | 0.037 |
| Lactate (mg/dL) | 1.80 [1.30,2.20] | | 2.20 [1.50,3.20] | <0.001 |
| T-CO_2_ (mEq/L) | 25.00 [25.00,28.00] | | 25.00 [20.00,28.00] | <0.001 |
| pH (units) | 7.37 (0.07) | | 7.34 (0.11) | <0.001 |
| PaO_2_ (mmHg) | 97.76 (20.57) | | 89.58 (22.54) | <0.001 |
| SaO_2_ (%) | 95.77 (5.99) | | 94.54 (7.18) | <0.001 |
| PaCO_2_ (mmHg) | 43.03 (9.39) | | 43.21 (12.36) | 0.561 |
| AG (mEq/L) | 14.70 (3.59) | | 16.00 (4.36) | <0.001 |
| BE (mEq/L) | 1.00 [0.00,2.00] | | 0.00 [-5.00,1.00] | <0.001 |
| Bicarbonate (mg/dL) | 24.61 (4.87) | | 22.98 (5.70) | <0.001 |
| Potassium (mEq/L) | 4.23 (0.72) | | 4.37 (0.86) | <0.001 |
| Sodium (mEq/L) | 138.20 (4.68) | | 137.65 (5.62) | <0.001 |
| Chloride (mEq/L) | 102.34 (6.14) | | 101.83 (6.79) | 0.007 |
| T-Calcium (mEq/L) | 8.56 (0.74) | | 8.35 (0.90) | <0.001 |
| Magnesium (mg/dL) | 2.00 (0.38) | | 2.03 (0.39) | 0.012 |
| Phosphate (mg/dL) | 3.60 [3.00,4.10] | | 3.80 [3.10,4.73] | <0.001 |
| INR | 1.30 [1.10,1.50] | | 1.40 [1.20,1.80] | <0.001 |
| APTT (s) | 31.40 [27.70, 39.00] | | 31.50 [27.50, 39.20] | 0.683 |
| PT (s) | 14.10 [12.40,17.50] | | 15.50 [13.20,21.90] | <0.001 |
| **Comorbidities (n%)** |  | |  |  |
| AF | 2221 (27.0) | | 342 (28.3) | 0.365 |
| T2DM | 1294 (15.7) | | 172 (14.2) | 0.192 |
| Hypertension | 2674 (32.5) | | 338 (28.0) | 0.002 |
| p-MI | 672 (8.2) | | 78 (6.5) | 0.045 |
| VA | 689 (8.4) | | 166 (13.7) | <0.001 |
| AKI | 2696 (32.8) | | 734 (60.8) | <0.001 |
| CLD | 2002 (24.4) | | 314 (26.0) | 0.231 |
| MT | 578 (7.0) | | 93 (7.7) | 0.435 |
| CKD | 6165 (75.0) | | 937 (77.6) | 0.059 |
| Anemia | 3805 (46.3) | | 575 (47.6) | 0.414 |

Values are mean + SD, n (%), or median (IOR).

HR, Heart rata; RR, Respiratory rate; SBP, Systolic blood pressure; DBP, Diastolic blood pressure; MAP, Mean arterial pressure; T, Temperature; RBC, Red blood cell; WBC, White blood cell; MCV, Mean corpuscular volume; MCH, Mean corpuscular hemoglobin; MCHC, Mean corpuscular hemoglobin concentration; ALT, Alanine transaminase; AST, aspartate aminotransferase; TB, Total Bilirubin; AP, Alkaline phosphatase; BUN, blood urea nitrogen; T-CO_2_, Total carbon dioxide; pH, potential of hydrogen; PaO_2_, arterial partial pressure of oxygen; PaCO_2_, arterial partial pressure of carbon-dioxide; SaO_2_, arterial oxygen saturation; AG, anion gap; BE, base excess; INR, International Normalized Ratio; PT, prothrombin time; APTT, activated partial prothrombin time; AF, atrial fibrillation; DM, Diabetes Mellitus; p-MI, previous myocardial infarction; VA, ventricular arrhythmias; AKI, acute kidney injury, CLD, chronic lung disease; MT, malignant tumor; CKD, chronic kidney diseases.

Supplementary table 5. Univariate logistic regression analysis results and multivariate logistic regression analysis results of all variables

| Variable | Odd Ratio | 95% CI | *P-value* | Odd Ratio (95%CI) | *P-value* |
| --- | --- | --- | --- | --- | --- |
| DBP/10 (mmHg) | 0.943 | 0.9182-0.9693 | ＜ 0.001 | 0.8783 (0.2677-2.8818) | 0.8305 |
| AG (mEq/L) | 1.094 | 1.0821-1.1062 | ＜ 0.001 | 1.02 (1.0027-1.0377) | 0.0234 |
| Age/10 (years) | 3.942 | 3.1456-4.9388 | ＜ 0.001 | 7.7534 (5.9472-10.1081) | ＜ 0.001 |
| Albumin (mg/dL) | 0.333 | 0.303-0.3666 | ＜ 0.001 | 0.4654 (0.4173-0.519) | ＜ 0.001 |
| ALT/100 (IU/L) | 1.067 | 1.0481-1.0852 | ＜ 0.001 | 0.9723 (0.9411-1.0045) | 0.0907 |
| AP/100 (IU/L) | 1.312 | 1.2511-1.3762 | ＜ 0.001 | 1.1521 (1.0937-1.2137) | ＜ 0.001 |
| APTT/10 (s) | 1.017 | 0.9996-1.0337 | 0.055 | - |  |
| SBP/10 (mmHg) | 0.908 | 0.8904-0.9252 | ＜ 0.001 | 0.903 (0.4984-1.6361) | 0.7365 |
| AST/100 (IU/L) | 1.063 | 1.0481-1.0786 | ＜ 0.001 | 1.0137 (0.9926-1.0352) | 0.2057 |
| Bicarbonate (mg/dL) | 0.946 | 0.9379-0.9543 | ＜ 0.001 | 1.009 (0.9978-1.0203) | 0.1139 |
| BUN (mg/dL) | 1.001 | 1.0009-1.0019 | ＜ 0.001 | 0.9996 (0.9989-1.0003) | 0.2650 |
| Creatinine (mg/dL) | 1.300 | 1.2458-1.3558 | ＜ 0.001 | 0.9718 (0.9125-1.035) | 0.3735 |
| Glucose (mg/dL) | 1.001 | 1.0006-1.0018 | ＜ 0.001 | 0.9995 (0.9987-1.0002) | 0.1371 |
| Hematocrit % | 0.980 | 0.9737-0.987 | ＜ 0.001 | 1.0081 (0.974-1.0434) | 0.6469 |
| Hemoglobin (g/dL) | 0.925 | 0.9067-0.9434 | ＜ 0.001 | 0.9968 (0.8924-1.1135) | 0.9554 |
| HR/10 (minˉ^1^) | 1.118 | 1.0949-1.142 | ＜ 0.001 | 1.0476 (1.0213-1.0745) | 0.0003 |
| INR | 1.253 | 1.202-1.307 | ＜ 0.001 | 1.0504 (0.968-1.1397) | 0.2382 |
| Lactate (mg/dL) | 1.312 | 1.2829-1.3427 | ＜ 0.001 | 1.1759 (1.1436-1.2091) | ＜ 0.001 |
| Magnesium (mg/dL) | 1.194 | 1.0656-1.3385 | 0.002 | 1.144 (1.0087-1.2975) | 0.0362 |
| MAP/10 mmhg | 0.899 | 0.8742-0.9238 | ＜ 0.001 | 1.2034 (0.2027-7.1449) | 0.8386 |
| MCH (pg) | 1.000 | 0.9828-1.0171 | 0.982 | - |  |
| MCHC (%) | 0.881 | 0.8579-0.9037 | ＜ 0.001 | 0.9451 (0.9012-0.9911) | 0.0199 |
| MCV (fL) | 1.007 | 1.0005-1.0135 | 0.035 | 0.9976 (0.9899-1.0053) | 0.5362 |
| PaCO_2_ (mmHg) | 1.000 | 0.9952-1.0038 | 0.815 | - |  |
| Phosphate (mg/dL) | 1.315 | 1.2695-1.3614 | ＜ 0.001 | 1.1216 (1.0704-1.1752) | ＜ 0.001 |
| Platelet/10 (k/μL) | 0.984 | 0.9786-0.9884 | ＜ 0.001 | 0.9807 (0.9752-0.9862) | ＜ 0.001 |
| PaO_2_/10 (mmHg) | 0.855 | 0.8391-0.8711 | ＜ 0.001 | 0.9244 (0.9046-0.9445) | ＜ 0.001 |
| PT/10 (s) | 1.179 | 1.1321-1.2274 | ＜ 0.001 | 1.0284 (0.9496-1.1138) | 0.4910 |
| RBC (m/uL) | 0.811 | 0.7653-0.86 | ＜ 0.001 | 0.8915 (0.7587-1.0476) | 0.1631 |
| RR/10 (minˉ^1^) | 1.701 | 1.5945-1.8146 | ＜ 0.001 | 1.3524 (1.2508-1.4623) | ＜ 0.001 |
| SaO_2_ (%) | 0.976 | 0.9704-0.9819 | ＜ 0.001 | 0.9937 (0.9866-1.0007) | 0.0790 |
| T (°C) | 1.042 | 0.9911-1.0946 | 0.108 | 1.0367 (0.9822-1.0941) | 0.1906 |
| TB (mg/dL) | 1.149 | 1.1244-1.1747 | ＜ 0.001 | 1.0909 (1.0637-1.1188) | ＜ 0.001 |
| T-Calcium (mEq/L) | 0.745 | 0.7038-0.7878 | ＜ 0.001 | - |  |
| T-CO_2_ (mEq/L) | 0.940 | 0.932-0.9486 | ＜ 0.001 | - |  |
| WBC (k/uL) | 1.041 | 1.0337-1.0491 | ＜ 0.001 | 1.0144 (1.0055-1.0233) | 0.0015 |
| AF | 1.116 | 1.0173-1.2232 | 0.020 | 1.062 (0.9509-1.1861) | 0.2858 |
|  | Reference |  |  |  |  |
| AKI | 2.838 | 2.5935-3.1054 | ＜ 0.001 | 2.153 (1.9487-2.3787) | ＜ 0.001 |
|  | Reference |  |  |  |  |
| p-MI | 0.748 | 0.6279-0.8898 | 0.001 | 0.8644 (0.7142-1.0462) | 0.1346 |
|  | Reference |  |  |  |  |
| T2DM | 0.827 | 0.7368-0.9293 | 0.001 | 0.9135 (0.7998-1.0435) | 0.1827 |
|  | Reference |  |  |  |  |
| Gender | 0.953 | 0.8724-1.0411 | 0.286 | - |  |
|  | Reference |  |  |  |  |
| Hypertension | 0.751 | 0.6839-0.8237 | ＜ 0.001 | 0.94 (0.8432-1.048) | 0.2646 |
|  | Reference |  |  |  |  |
| VA | 1.591 | 1.39-1.8214 | ＜ 0.001 | 1.6273 (1.3944-1.8991) | 0.0000 |
|  | Reference |  |  |  |  |
| CLD | 1.085 | 0.9858-1.1935 | 0.096 | - |  |
|  | Reference |  |  |  |  |
| MT | 1.146 | 0.9634-1.3637 | 0.124 | - |  |
|  | Reference |  |  |  |  |
| CKD | 1.035 | 0.9321-1.1487 | 0.522 | - |  |
|  | Reference |  |  |  |  |
|  | 1.023 | 0.9786-1.0686 | 0.319 | - |  |
|  | Reference |  |  |  |  |
| Anemia | 1.026 | 0.9388-1.1217 | 0.570 | - |  |
|  | Reference |  |  |  |  |
| BE ＜-3/＞3 (mmol/L) | Reference |  |  |  |  |
| -3~3 (mmol/L) | 2.268 | 2.0733-2.4809 | ＜ 0.001 | 1.3368 (1.2013-1.4875) | ＜ 0.001 |
| pH ＜7.35/＞7.45 (units) | Reference |  |  |  |  |
| 7.35~7.45 (units) | 2.415 | 2.2081-2.6411 | ＜ 0.001 | 1.5149 (1.3636-1.6829) | ＜ 0.001 |
| Potassium ＜3.5/＞5.5 (mEq/L) | Reference |  |  |  |  |
| 3.5~5.5 (mEq/L) | 1.329 | 1.1877-1.4861 | ＜ 0.001 | 1.054 (0.9288-1.1961) | 0.4148 |
| Sodium ＜135/＞155(mEq/L) | Reference |  |  |  |  |
| 135~155 (mEq/L) | 1.622 | 1.4625-1.7988 | ＜ 0.001 | 1.2621 (1.1191-1.4234) | ＜ 0.001 |
| Chloride ＜135/＞155 （mEq/L) | Reference |  |  |  |  |
| 95~105（mEq/L） | 1.155 | 1.0551-1.2638 | 0.002 | 1.0016 (0.9038-1.1099) | 0.9755 |

HR, Heart rata; RR, Respiratory rate; SBP, Systolic blood pressure; DBP, Diastolic blood pressure; MAP, Mean arterial pressure; T, Temperature; RBC, Red blood cell; WBC, White blood cell; MCV, Mean corpuscular volume; MCH, Mean corpuscular hemoglobin; MCHC, Mean corpuscular hemoglobin concentration; ALT, Alanine transaminase; AST, aspartate aminotransferase; TB, Total Bilirubin; AP, Alkaline phosphatase; BUN, blood urea nitrogen; T-CO_2_, Total carbon dioxide; pH, potential of hydrogen; PaO_2_, arterial partial pressure of oxygen; PaCO_2_, arterial partial pressure of carbon-dioxide; SaO_2_, arterial oxygen saturation; AG, anion gap; BE, base excess; INR, International Normalized Ratio; PT, prothrombin time; APTT, activated partial prothrombin time; AF, atrial fibrillation; DM, Diabetes Mellitus; p-MI, previous myocardial infarction; VA, ventricular arrhythmias; AKI, acute kidney injury, CLD, chronic lung disease; MT, malignant tumor; CKD, chronic kidney diseases.

Supplementary table 6: Model Variable Score Table

| **Variable** | **β Value** | **OR Value** | **95% CI** | ***P* value** | **Rating Score** |
| --- | --- | --- | --- | --- | --- |
| Age (＞60 years) | 0.840 | 2.317 | 1.928-2.784 | ＜0.001^a^ | 8 |
| RR (＜8bpm /＞20bpm) | 0.347 | 1.414 | 1.240-1.614 | ＜0.001^a^ | 4 |
| PaO_2_ (＜60mmHg) | 0.437 | 1.548 | 1.222-1.961 | ＜0.001^a^ | 4 |
| Platelet (＜100k/μL /＞300k/μL) | 0.364 | 1.439 | 1.245-1.663 | ＜0.001^a^ | 4 |
| Albumin (＜3mg/dL) | 0.718 | 2.049 | 1.774-2.368 | ＜0.001^a^ | 7 |
| TB (＞2mg/dL) | 0.452 | 1.571 | 1.264-1.952 | ＜0.001^a^ | 5 |
| AP (＞180IU/L) | 0.459 | 1.583 | 1.271-1.973 | ＜0.001^a^ | 5 |
| Lactate mg/dL | 0.212 | 1.237 | 1.191-1.284 | ＜0.001 ^a^ | 2 |
| pH (＜7.35 units /＞7.45units) | 0.360 | 1.434 | 1.241-1.657 | ＜0.001^a^ | 4 |
| BE (＜-3 mmol/L /＞3 mmol/L ) | 0.391 | 1.478 | 1.279-1.709 | ＜0.001^a^ | 4 |
| Phosphate (mg/dL) | 0.212 | 1.236 | 1.079-1.417 | 0.002^a^ | 2 |
| VA | 0.406 | 1.501 | 1.229-1.834 | ＜0.001^a^ | 4 |
| AKI | 0.888 | 2.431 | 2.128-2.778 | ＜0.001^a^ | 9 |

Note: ^a^*P* value <0.05 was considered statistically significant.

RR, Respiratory rate; PaO_2_, arterial partial pressure of oxygen; TB, Total Bilirubin; AP, Alkaline phosphatase; pH, potential of hydrogen; BE, base excess; VA, ventricular arrhythmias; AKI, acute kidney injury.

Supplementary table 7: Feature Screening

| **Variable** | Logistic regression | Lasso regression  (Only 12 variables) | Lasso regression (Only 12 variables) |
| --- | --- | --- | --- |
| Age | **+** | **+** | **+** |
| RR | **+** | **+** | **+** |
| PaO_2_ | **+** | **+** | **+** |
| Platelet | **+** | **+** | **+** |
| Albumin | **+** | **+** | **+** |
| TB | **+** | **+** | **+** |
| AP | **+** | **+** | **+** |
| Lactate | **+** | **+** | **+** |
| T-CO_2_ | **-** | **+** | **-** |
| pH | **+** | **+** | **+** |
| AG | **-** | **+** | **-** |
| BE | **+** | **+** | **+** |
| Phosphate | **+** | **+** | **+** |
| INR | **-** | **+** | **-** |
| AKI | **+** | **+** | **+** |
| VA | **+** | **-** | **-** |

RR, Respiratory rate; PaO_2_, arterial partial pressure of oxygen; TB, Total Bilirubin; AP, Alkaline phosphatase; T-CO_2_, Total carbon dioxide; pH, potential of hydrogen; AG, anion gap; BE, base excess; INR, International Normalized Ratio; VA, ventricular arrhythmias; AKI, acute kidney injury.
